# A non-canonical Raf function is required for dorsal–ventral patterning during *Drosophila* embryogenesis

**DOI:** 10.1038/s41598-022-11699-3

**Published:** 2022-05-10

**Authors:** Jay B. Lusk, Ellora Hui Zhen Chua, Prameet Kaur, Isabelle Chiao Han Sung, Wen Kin Lim, Vanessa Yuk Man Lam, Nathan Harmston, Nicholas S. Tolwinski

**Affiliations:** 1 Division of Science, Yale-NUS College, Singapore, 138527 Singapore; 2 Program in Cancer and Stem Cell Biology, Duke-NUS Medical School, Singapore, 169857 Singapore; 3 Yale-NUS College Research Labs @ E6, E6, 5 Engineering Drive 1, #04-02, Singapore, 117608 Singapore

**Keywords:** Cell lineage, Epithelial-mesenchymal transition

## Abstract

Proper embryonic development requires directional axes to pattern cells into embryonic structures. In *Drosophila*, spatially discrete expression of transcription factors determines the anterior to posterior organization of the early embryo, while the Toll and TGFβ signalling pathways determine the early dorsal to ventral pattern. Embryonic MAPK/ERK signaling contributes to both anterior to posterior patterning in the terminal regions and to dorsal to ventral patterning during oogenesis and embryonic stages. Here we describe a novel loss of function mutation in the Raf kinase gene, which leads to loss of ventral cell fates as seen through the loss of the ventral furrow, the absence of Dorsal/NFκB nuclear localization, the absence of mesoderm determinants Twist and Snail, and the expansion of TGFβ. Gene expression analysis showed cells adopting ectodermal fates much like loss of Toll signaling. Our results combine novel mutants, live imaging, optogenetics and transcriptomics to establish a novel role for Raf, that appears to be independent of the MAPK cascade, in embryonic patterning.

## Introduction

A central question of embryonic development is how fates are assigned to thousands of cells within the three-dimensional structure of an egg or embryo. Along the dorsal–ventral axis, the early *Drosophila* embryonic cell fates are determined by the Toll and Dpp/TGFβ pathways, where cells with active Toll adopt a ventral cell fate, and cells with active TGFβ adopt a dorsal cell fate^1–3^. EGF signaling patterns the neuroectoderm in more lateral cells^4^, and the combination of EGF, TGFβ and Toll signals establish the dorsal–ventral (D/V) axis in early embryos^4–7^.

The EGF signaling pathway is one of the most studied signal transduction cascades in development and disease. The signaling cascade begins with *Epidermal Growth Factor (EGF) receptor (EGFR* or *Der* in *Drosophila)* a transmembrane receptor tyrosine kinase which, among other functions, activates the small GTPase Ras, initiating the Mitogen Activated Protein Kinase (MAPK) cascade through the activation of the Raf kinase^8,9^. *EGFR*’s *faint little ball* phenotype, which is characterized by the loss of ventral and terminal structures, highlights the importance of the pathway and its components in assigning cell fates^10–13^.

The Raf kinase acts downstream of multiple receptor tyrosine kinases in establishing *Drosophila*’s body axes. For example, Raf’s kinase activity downstream of the *torso* receptor tyrosine kinase (RTK) is essential for patterning anterior and posterior termini in the embryo^14,15^. In follicle cells, the *Torso-like* ligand is only expressed in polar populations. Thus, during embryogenesis, Torso RTK patterning is localized to terminal regions. During early development of the embryo, the ligand binds to Torso and activates Raf, leading to transcriptional activation of zygotic genes *tailless*, *huckebein*, and repression of *bicoid* target genes, such as *hunchback* and *orthodenticle*. The genes then determine cellular fates at the anterior/posterior termini, giving rise to specialized structures at the terminal regions of the larva^16^. Embryos lacking either maternal *Raf* or *Tor* activities demonstrate significant aberrations in anteroposterior patterning, including loss of hindgut and posterior midgut structures, derivatives of abdominal segments 8–10, specific head skeletal structures, and Malphigian tubules^17–19^.

Raf also acts downstream of EGFR to determine ventral ectodermal and lateral fates during embryogenesis^20–22^. Without EGFR activity, embryos will display the *faint little ball* phenotype, characterized by the presence of only the dorsal hypoderm in cuticles and a loss of ventral and terminal structures^10–13^. As Raf works downstream of EGFR in developing the ventral ectoderm, a constitutively active Raf can bypass defective *EGFR* and rescue the *faint little ball* phenotype^15^.

Raf also plays other roles in development. For instance, Raf acts upstream of *seven-in-abstentia* (*sina*) and downstream of Ras and receptor tyrosine kinase *sevenless* (*sev*) in the Sev pathway to establish R7 photoreceptor cells in the eye^23–25^. Constitutively activated Raf can induce R7 development in the *sevenless* phenotype that lacks R7 in every ommatidium of the eye^24^. After development, EGFR signaling plays a multitude of roles ranging from wound healing to homeostasis in both vertebrates and *Drosophila*^26–28^.

Raf is essential for establishing dorsoventral cell fates during oogenesis. During oogenesis, the asymmetrically anchored oocyte nucleus defines a presumptive dorsal region that contains high levels of Gurken, a transforming growth factor (TGF-α) ligand that activates EGFR in the dorsal oocyte follicular epithelium^29–32^. The activating signal from Gurken is then transduced through the classical MAPK cascade, leading to the transcription of a variety of target genes, such as *rhomboid*^33^. Rhomboid is then apically localized in the dorsal follicle cells on the anterior side of the egg chamber. It is required to induce dorsal cell fates in the oocyte and consequently in the embryo^34^. *Rhomboid* also activates another EGFR ligand, Spi, through an intracellular cleavage mechanism^33^. At the maximum level of EGF signaling, located at the dorsal midline, the EGF repressor *argos* is expressed, with the end result being two stripes of *rhomboid* expression which surround the dorsal midline, thereby generating a complex dorsal–ventral pattern in the follicle cells which will later influence embryonic development^35,36^.

The Gurken activated EGF pathway represses *pipe* in the dorsal region follicle layer of the oocyte^31,37^. When the egg is fertilized, Pipe in the ventral region of the embryo facilitates a proteolytic cascade that results in the release of Spätzle. Spätzle then activates the Toll pathway receptor and delimits and orients the dorsoventral axis of the embryo^38^. Above all, EGF signaling in the maternal follicle cells is required to repress ventral cell fates and is sufficient to induce dorsal cell fates in the oocyte. More specifically, contacts between the follicle cells and the oocyte during oogenesis can cause limited activation of EGFR in the oocyte, which represses ventral cell fates in the developing oocyte^33,39,40^. This initial dorsal–ventral polarity of the oocyte later becomes important in the dorsal–ventral patterning of the fertilized embryo.

Once the Toll receptor has been activated by Spätzle, the *Drosophila* homologue of myeloid differentiation primary response 88 (*dMyD88* or *krapfen (kra))* binds directly to Toll and interacts with the death domain of a downstream protein called Tube^41,42^. Tube, the orthologue of mammalian Interleukin-1 receptor associated kinase 4 (IRAK-4), is a short protein containing an N-terminal death-domain, and a C-terminal Tube repeat domain that participates in protein–protein interactions^43^. Unlike mammalian IRAKs, Tube does not have a kinase domain and relies instead on Pelle kinase to transduce the Toll signal. The Tube/MyD88 complex binds to Pelle, Pelle is autophosphorylated, which is required for Toll signal transduction^44,45^. IκB-α (*Drosophila* Cactus), in the absence of Toll signaling, binds to RelA/NF-κB (*Drosophila* Dorsal) sequestering NF-κB in the cytoplasm, leading to NF-κB degradation^46^. Phosphorylation of IκB-α leads to its degradation, which releases NF-κB, allowing NF-κB to translocate to the nucleus to initiate transcription^47^. In mammals, the IκB kinase (IKK) complex is composed of two kinases (IKKα and IKKβ) and a regulatory NEMO subunit^48^. The canonical (pro-inflammatory response) pathway is activated by phosphorylation of IKKβ, and the non-canonical pathway (associated with lymphoid cell proliferation) is activated by phosphorylation of IKKα, which itself is activated by a distinct upstream kinase called the NF-κB-inducing kinase (NIK), which is a member of the Map Kinase Kinase Kinase family (MAP3K14)^49,50^. The *Drosophila* Toll pathway uses a different IKK complex^51^, where the kinase Pelle can fulfil the functional role of IKKβ^52^.

Activation of Toll followed by the phosphorylation and degradation of Cactus/IκB^38,53^ frees Dorsal/NFκB to enter the nucleus and begin transcription^54–57^. Once inside the nucleus, Dorsal activates or represses a wide variety of genes, including key developmental regulators *decapentaplegic, zerknüllt, twist,* and *snail,* which pattern much of the rest of the embryo^7,58^. Although the centrality of Toll signaling for D/V patterning has long been established, not much is known about signaling cross talk between the Toll pathway and the EGFR/Ras/Raf pathway in early embryogenesis. The canonical functions of Raf require the MAPK cascade, but there are some examples where Raf functions independently^59–62^. Here, we turn to examining the role Raf plays in early patterning events that do not coincide with previously defined domains of MAPK activity by analyzing new mutants.

## Results

### A dorsalizing *Raf* mutation

**Figure 1:**
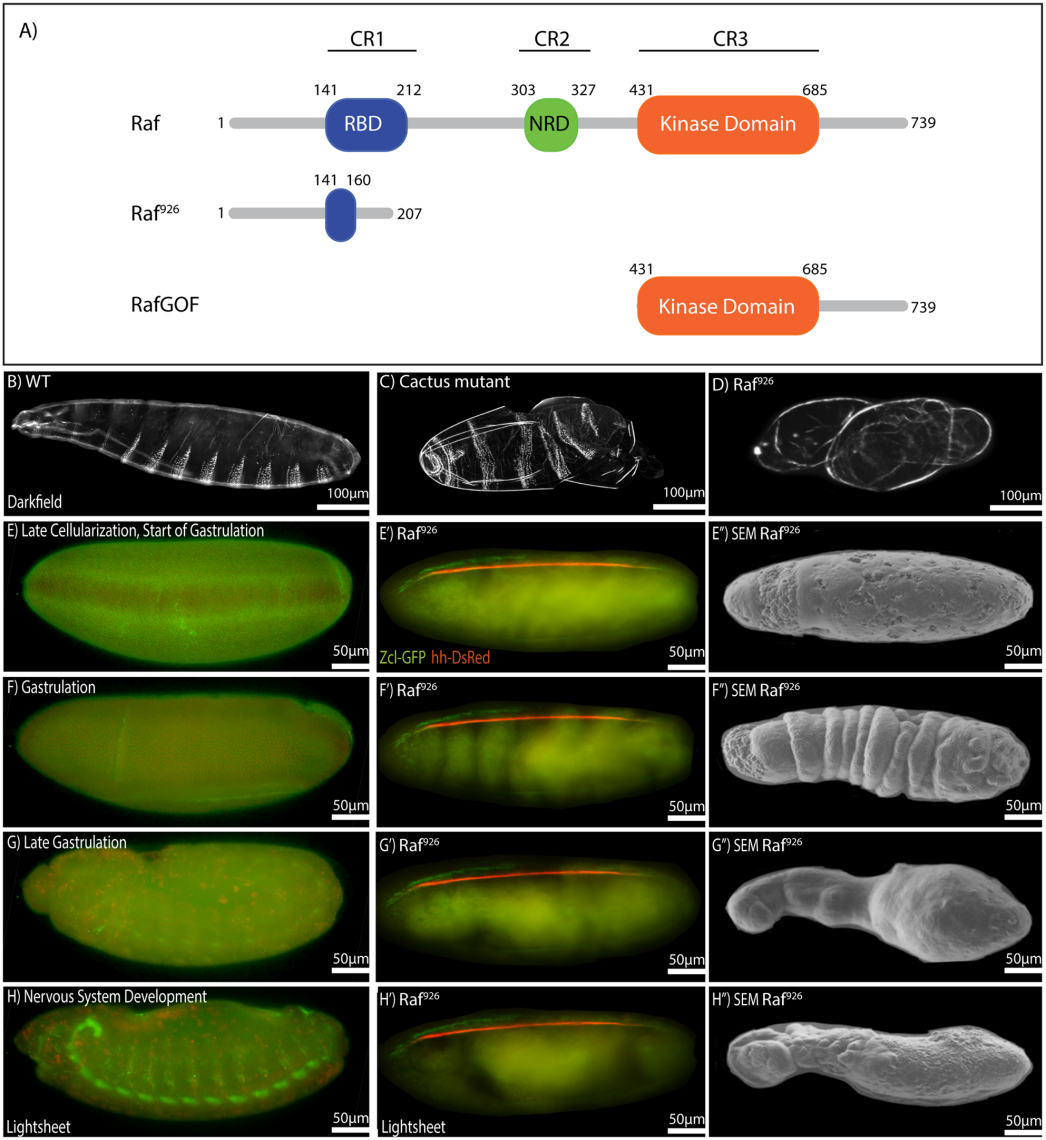
Schematic illustration, cuticular phenotype and developmental progression of *Raf*^*926*^ mutant embryo. (**A**) Domain organization of *Drosophila Raf, Raf*^*926*^ and *Raf*^*GOF*^. Raf protein contains the Ras-binding domain (RBD) in conserved region 1 (CR1), negative regulatory domain (NRD) in CR2 and protein kinase domain in CR3, with a total length of 739 amino acids. Raf^926^ comprised of an altered RBD with 207 amino acids in total due to a deletion of 17 nucleotides which resulted in a frameshift and premature stop codon. Raf^GOF^ contains the kinase domain only, with total length of 309 amino acids. Dark field micrographs of the cuticles of wild type embryo showing normal distribution of ventral denticles and dorsal hair (**B**), a *cactus*^*1*^ mutant embryo showing strongly ventralized phenotype with ventral denticles expression among the dorsal hair (**C**) and *Raf*^*926*^ mutant embryo showing a strongly dorsalized phenotype with elongated, tube-like, twisted body entirely covered by dorsal hair (**D**). (**E**–**H**) Still images of developmental stages of a wild-type embryo to be compared to still images of developmental stages of *Raf*^*926*^ embryo from lightsheet (**E′**–**H′**) and scanning electron microscope (SEM) (**E″**–**H″**). *Raf*^*926*^ embryos develop normally up to cellularization stage. Defective gastrulation is characterized by frequent twisting, elongation of twisted segments, fusions of segments into three main sacs, followed by tissue death. Embryo was visualised with Utrophin-GFP and Histone-RFP, with anterior to the left. Stills were extracted from Video 3.

To identify genes involved in D/V patterning, we screened a new library of molecularly defined mutations for defects in axis formation^63^. ~ 2000 X-chromosome mutants were independently crossed to generate maternally and zygotically mutant embryos and screened for patterning defects by cuticle phenotypes^64–66^. We discovered a strong dorsalizing mutation (926, Fig. 1D) that mapped through complementation analysis (using molecularly defined duplications covering the X-chromosome P[acman]^67^) to a chromosomal region containing the *Raf* gene. Molecular analysis showed that the *Raf*^*926*^ DNA sequence contained a deletion of 17 nucleotides beginning at the 479th nucleotide of the protein-coding region. This lesion created a frame shift in the coding sequence, leading to a premature stop codon before the second and third conserved domains, in addition to deleting the majority of the first conserved domain (Fig. 1A). This deletion was predicted to be a strong loss of function allele as the stop codon eliminates most of the Ras-binding domain (RBD) in conserved region 1 (CR1), the negative regulatory domain (NRD) in CR2 and the protein kinase domain in CR3. We were unable to detect mRNA or to make cDNA, likely as a result of nonsense mediated decay^68^. The stop codon is in a similar location to the strong, amorphic Class 1 alleles of *Raf* reported^18^. To confirm that the allele was a loss of function allele and not a dominant negative allele, we cloned genomic DNA from *Raf*^*926*^ into an overexpression construct tagged with mCherry. Overexpression did not show a phenotype in embryonic patterning and development proceeded normally (Combined Videos 1, Video 1). Although the textbook definition of disrupted EGF signaling mentions dorsalization (*Drosophila* Redbook^13^), previous analyses of Raf mutants did not focus on the ventral most cell fates. We proceeded to test more Raf mutants such as the point mutants *Raf*^*A*^ and *Raf*^*B*^, but these did not produce embryos suggesting oogenesis was disrupted^63,69^. To overcome this limitation, we turned to a CRISPR/Cas9 approach where maternally expressed Cas9 drives gRNA directed mutations in the *Raf* gene^70^. As the CRISPR/Cas9 system introduces mutations randomly and at different developmental timepoints, we observed a variety of phenotypes including dorsalized embryos as shown by the presence of dorsal hairs (S. Fig. 1A–F) as well as other patterning phenotypes, which likely depend on the timing of the induced genetic lesion.

The prototypical dorsalizing mutations such as *dorsal* and *Toll* show an elongated, tube-like embryo that twists within the eggshell and lacks all ventral features such as denticles^2,71^. *Raf*^*926*^ showed a similar highly-dorsalized phenotype where the embryos appear to twist around themselves and fail to develop ventral structures such as denticles (Fig. 1D). Although the twisting phenotype could vary in severity, the maternal effect mutation was 100% penetrant and could not be rescued by paternal contribution. As EGF is involved in oocyte patterning, we inspected ovarioles and egg morphology to determine if any abnormalities were present^32,72^. We observed that ovarioles were unaffected and oocytes developed standard shape and polarity including normal dorsal appendages. We concluded that the embryonic phenotype was not due to defects in oogenesis, but rather was due to altered signaling in embryogenesis.

To observe early developmental stages of these mutants, we used a live imaging approach through lightsheet microscopy^73,74^. *Raf*^*926*^ embryos appear morphologically normal through the cellularization stage (Fig. 1E) but begin to show abnormalities during the first process of gastrulation (Fig. 1F), where no ventral furrow is formed. Without a ventral furrow, cells cannot enter the interior of the embryo to form the mesoderm, and the cell movements of gastrulation in dorsalized embryos become unpredictable (Fig. 1G,H, Combined Video 1, Videos 2 and 3 compared to wild-type Video 4). Nevertheless, we looked at the various stages from live imaging and compared these to fixed embryos in scanning electron microscopy to show the various stages of tissue movements (Fig. 1E–H, Video 3). These showed a twisting and elongation of the ectodermal tissue (Fig. 1E–H) without the ventral denticle structures that would be seen in a ventralizing mutation such as *cactus* (Fig. 1C). It is important to point out that developmental stages in fixed embryos could not be readily established as the normal hallmarks of gastrulation such as ventral and cephalic furrows and germband extension were disrupted.

**Figure 2:**
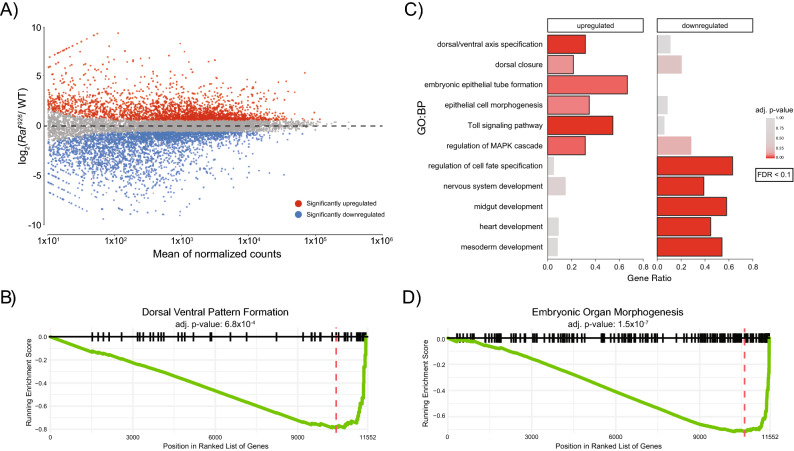
Transcriptional profiling of WT, *Raf*^*926*^ embryos identifies significant differences in the expression of genes involved in Dorsal/Ventral patterning, organogenesis and the Toll pathway. (**A**) MA-plot shows a large number of genes are significantly differentially expressed between *Raf*^*926*^ mutant and WT embryos (three biological replicates per condition). (**B**) GSEA identifies that genes annotated as involved in dorsal–ventral pattern formation are altered in *Raf*^*926*^ mutants. (**C**) GO Biological Process (GO:BP) enrichment analysis identifies that the significant up- and downregulated genes are enriched for distinct processes. (**D**) GSEA identifies that genes are involved in embryonic organ morphogenesis and perturbed in *Raf*^*926*^ mutants.

The primary ventral feature in early stages of gastrulation is the ventral furrow. It is one of the first morphogenetic events of gastrulation and leads to the internalization of mesodermal precursors. Gastrulation starts immediately after cellularization and results in a furrow along the ventral midline^75^. Apical constriction occurs in a 12 cell-wide region surrounding the ventral midline, and these midventral cells then drag three rows of cells on each side towards the ventral midline. As the midventral cells continue to contract, neighboring lateral cells are continuously dragged into the furrow^76,77^. To determine whether *Raf* loss-of-function could cause disruptions to ventral development during pre- to early gastrulation, we looked at brightfield Videos to follow development of the ventral side of *Raf*^*926*^ embryos starting from before stage 5 to stages 6 or 7. Both wildtype and *Raf*^*926*^ appeared similar during cellularization (Combined Video 2, Compare Video 5 to Video 6, Still Images in S. Fig. 2A–D), but after cellularization, the wildtype embryo formed a clear ventral furrow whereas *Raf*^*926*^ did not (Video 6, Still Images in S. Fig. 2A–D).

Given the early role of EGFR signaling in terminal structure patterning (downstream of *torso* and *torsolike*^19,78^), we investigated whether disrupted anterior to posterior (A/P) patterning could explain the loss of the ventral furrow. *Raf* mutants show a loss of terminal A/P structures as well as D/V patterning defects^17,18,79^. To confirm that the lack of ventral furrow was not due to anteroposterior defects caused by Raf’s other role, we used the triple-mutant *bicoid*, *nanos*, *torso-like* (*bnt*) which lacks anteroposterior patterning^80^. Much like wildtype embryos, *bnt* triple mutants developed a ventral furrow (Video 7, Still Images in S. Fig. 2A–D). The presence of ventral furrows in *bnt* and the absence of ventral furrows in *Raf*^*926*^ mutants suggested a role for Raf in ventral morphogenic events that cannot be accounted for by abnormal anterior/posterior patterning.

### Transcriptional analysis of ***Raf***^***926***^ mutants identifies changes in key D/V processes and pathways

**Figure 3:**
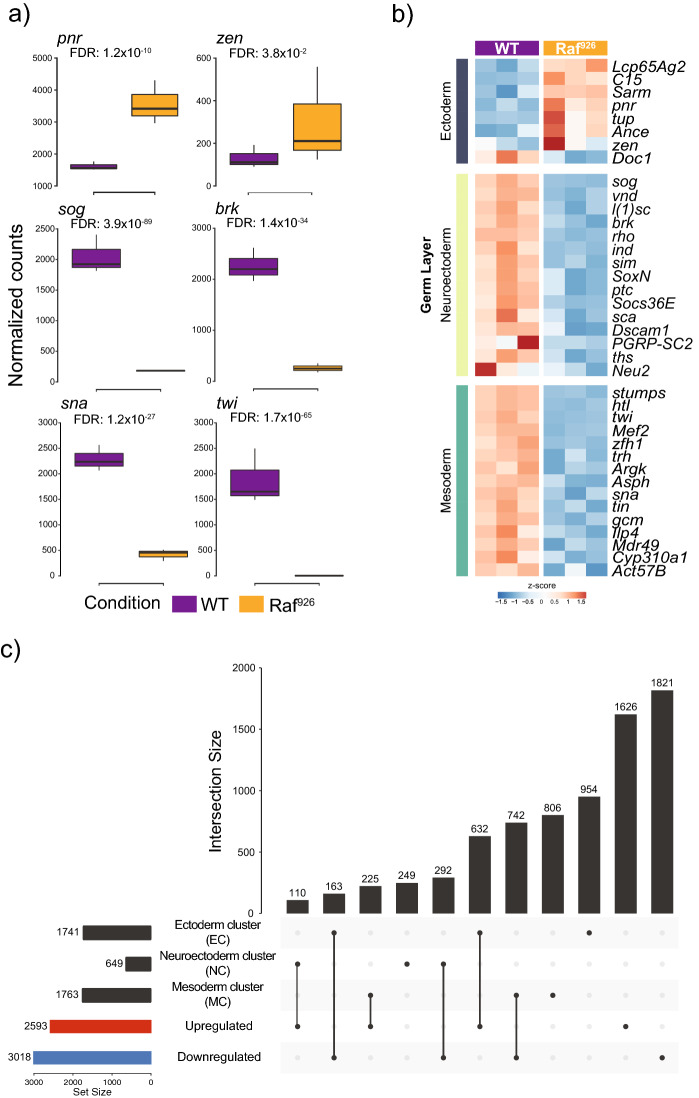
Comparison of expression changes in observed *Raf*^*926*^ mutants with key germ layer marker genes. (**A**) Expression profiles of key germ layer marker genes in WT and *Raf*^*926*^ mutant embryos. (**B**) *Raf*^*926*^ mutants show upregulation of ectoderm marker genes, along with downregulation of neuroectoderm and mesoderm marker genes. (**C**) Upset plot showing the overlap between differentially expressed genes in *Raf*^*926*^ mutant with clusters of gene expression changes observed after generating embryos with only one germ layer after perturbing the Toll pathway (see S. Fig. 3D).

The EGFR signaling pathway has not previously been associated with ventral cell fates and previous studies have not detected phosphorylated ERK in ventral cells^8,81^. *Raf*^*926*^ mutants do not make a ventral furrow preventing standard staining or in situ RNA hybridization from being informative. To overcome this limitation, we investigated the transcriptional changes occurring in *Raf*^*926*^ mutants during early embryogenesis stages (Staged from 0 to 12 h post egg laying). We collected and sequenced mRNA from *Raf*^*926*^ maternally mutant embryos and compared their transcriptional profiles to wild type (WT) embryos (S. Fig. 3A). As expected, total Raf expression was reduced by more than half (S. Fig. 3B) as expected for embryos maternally deficient for Raf crossed to hemizygous males. We found that 5611 genes were significantly differentially expressed (FDR < 10%, fold change > 1.5), with 2593 genes upregulated in *Raf*^*926*^ mutant embryos and 3018 downregulated compared to WT (Fig. 2A, Supplemental Table 1).

The expression of genes involved in dorsal–ventral pattern formation was significantly dysregulated in *Raf*^*926*^ mutants (Fig. 2B). Genes that were upregulated in *Raf*^*926*^ mutants were enriched for processes associated with ectoderm, including epithelial cell morphogenesis (FDR < 10%, Fig. 2C). The Toll pathway is required for the formation of ventral cells and TGFβ for dorsal cell fates. Surprisingly, we observed that components of the Toll signal transduction pathway were upregulated in *Raf*^*926*^ mutants (Fig. 2C, S. Fig. 3C), but genes believed to be downstream of pathway activation were downregulated (see below). Whereas the set of downregulated genes was enriched for processes related to nervous system and mesoderm development (FDR < 10%, Fig. 2C). In addition, GSEA identified genes involved in embryonic organ morphogenesis as dysregulated in *Raf*^*926*^ embryos (Fig. 2D). These results are consistent with the lack of ventral furrow formation as many internal structures would be missing without the generation of a mesoderm in *Raf*^*926*^ mutants.

### Expression changes in ***Raf***^***926***^ mutants are similar to those observed in Toll pathway mutants

**Figure 4:**
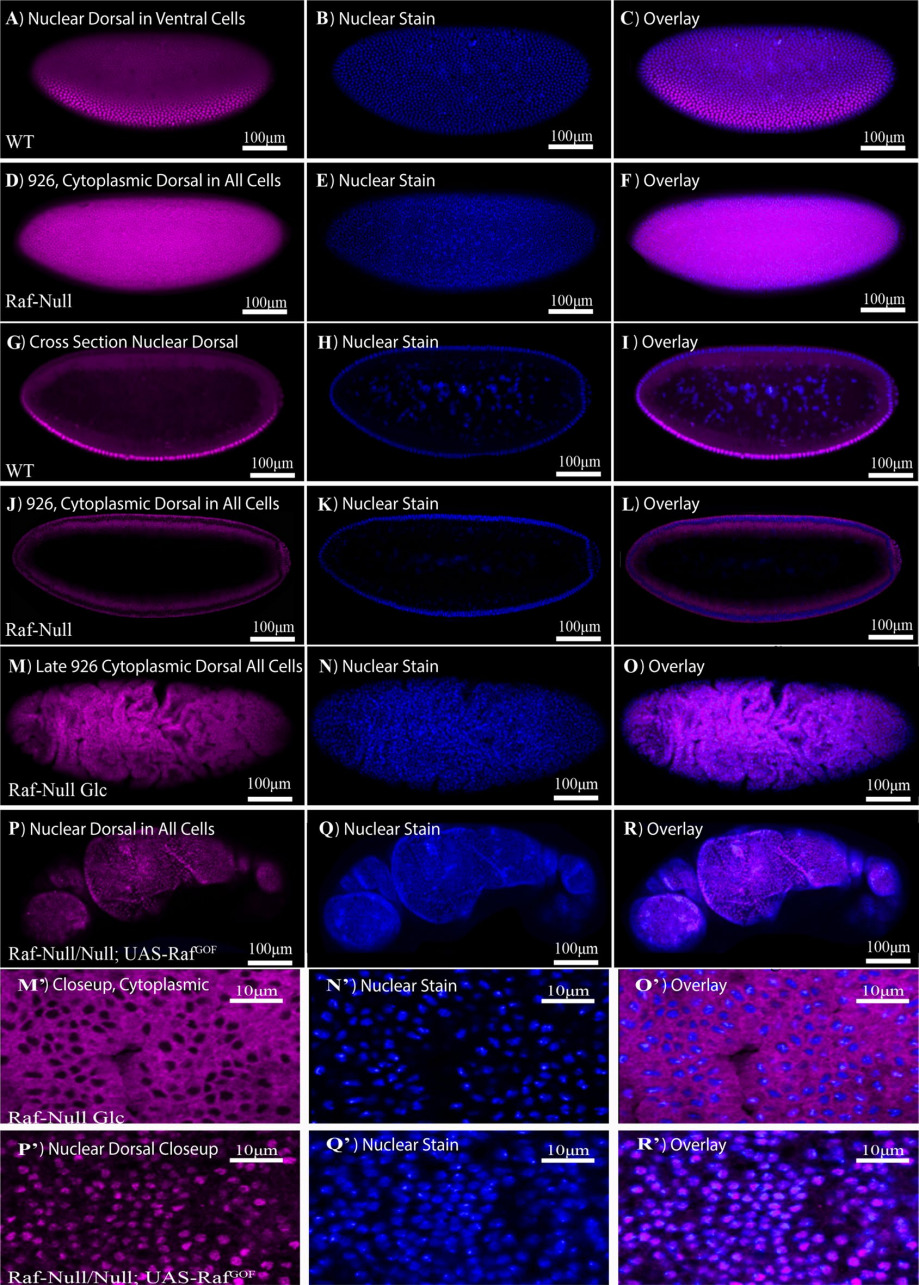
Dorsal nuclear localization in WT, *Raf*^*926*^ embryo and *Raf*^*926*^ embryo expressing *Raf*^*GOF*^. Surface view of the lateral orientation of the wildtype embryo (**A**) undergoing cellularization with a nuclear Dorsal gradient along the dorsal–ventral axis, (**B**) shows the corresponding nuclear stain and (**C**) a merged image. Surface view of the lateral orientation of the *Raf*^*926*^ embryo (**D**) undergoing cellularization with complete exclusion of Dorsal from the nuclei. (**E**) The corresponding nuclear stain and (**F**) a merged image of both. Cross-sectional view of the lateral orientation of the (**G**) wildtype embryo showing the gradient nuclear localization of Dorsal along the dorsal–ventral axis, (**H**) shows the corresponding nuclear stain and (**I**) a merged image of both. Cross-sectional view of *Raf*^*926*^ embryo (**J**) showing the complete exclusion of Dorsal from the nucleus, (**K**) showing the corresponding nuclear stain and (**L**) a merged image of both. Surface view of (**M**, **M′**) *Raf*^*926*^ later stage embryo showing abnormality in development after cellularization and exclusion of Dorsal from the nucleus. The corresponding nuclear stain (**N**, **N′**) and merged images (**O**, **O′**). (**P**, **P′**) *Raf*^*926*^ embryo expressing *Raf*^*GOF*^ showing dorsal nuclear localization. (**Q**, **Q′**) shows the corresponding nuclear stain and (**R**, **R′**) the merged image.

To better understand how the transcriptional changes that were observed in *Raf*^*926*^ mutants reflected differences in germ layer formation, we compared the set of differentially expressed genes with those identified in two distinct datasets investigating germ layer formation during early embryonic development. Stathopoulous et al*.* investigated gene expression changes in three distinct early embryonic regions as defined by levels of Toll signaling^82^. They defined the region with high and medium Toll signalling as mesoderm and neuroectoderm respectively, while the dorsal ectoderm was characterized as the region lacking Toll signalling. Marker genes were subsequently identified for each of these regions. We investigated the effect of *Raf*^*926*^ on the expression of these three cohorts of dorsal/ventral patterning markers. The majority of Dorsal targets (15/20) in the mesoderm were significantly downregulated in *Raf*^*926*^ mutants (Fig. 3B, S. Fig. 3C), including the key mesoderm determinants twist (*twi*) and snail (*sna*) (Fig. 3A). We next looked at Dorsal targets in the neuroectoderm and found that 15/20 targets were significantly downregulated in the *Raf*^*926*^ mutants (Fig. 3B, S. Fig. 4B). In particular, the Dpp pathway regulators *sog* and *brk* (Fig. 3A), as well as the neuroectoderm determining transcription factors intermediate neuroblasts defective (*ind*) and ventral nervous system defective (*vnd*) were downregulated. The third set corresponds to genes that are repressed by Dorsal and as such are expressed on the dorsal side of the embryo which gives rise to the ectoderm. In dorsalized embryos, a condition where no Toll signal is present, these genes increase in expression^82,83^. We investigated this set of genes and found that 7/13 were significantly upregulated in *Raf*^*926*^ mutants (Fig. 3B, S. Fig. 4A), including key transcription factors involved in dorsal cell fate determination (i.e. pannier (*pnr*) and zerknullt (*zen*), Fig. 3A).

Embryos can be generated which consist of either dorsal ectoderm, neuroectodermal or mesodermal precursor cells using specific mutant lines; gd^7^, Toll^rm9^/^rm10^ and Toll^10b^ respectively. Previously, Koenecke et al*.* characterized the epigenetic and transcriptional landscapes of these mutant embryos^83,84^. Re-analysis of their RNA-sequencing dataset identified 4173 significantly differentially expressed genes (FDR < 10%) which clustered into three distinct groups, each associated with distinct germ layers/mutant lines (S. Fig. 4D,E). We compared each of these clusters with the set of significantly differentially expressed genes observed in the *Raf*^*926*^ mutant RNA-seq analysis (Fig. 3C). There was a significant overlap (N = 629, p < 5 × 10^–4^) between genes upregulated by *Raf*^*926*^ and those genes located in the cluster of genes specifically upregulated in the dorsal ectoderm (EC). The set of genes downregulated by *Raf*^*926*^ significantly overlapped with genes upregulated in neuroectoderm (NC) (N = 292, p < 5 × 10^–4^), and the mesoderm (MC) (N = 739, p < 5 × 10^–4^) (S. Fig. 4A–C,F). This supports that *Raf*^*926*^ mutant embryos are transcriptionally similar to the dorsalized embryos that are generated by perturbing the Toll pathway.

Overall, the transcriptional analysis showed that *Raf*^*926*^ mutants recapitulated the effect of strong loss of function mutants in the Toll pathway with most key mesoderm and neuroectoderm determining genes being significantly downregulated and ectoderm determinants being significantly de-repressed. This pattern of expression changes supports that these are dorsalized embryos, which lack both the mesoderm and neuroectoderm germ layers and are essentially empty epidermal pouches. The surprising finding was that despite upregulation of Toll signal transduction pathway genes, the expected transcriptional targets of Dorsal were downregulated suggesting Raf is involved in the pathway or functions in parallel to Dorsal, but is required for full pathway activity.

### Effect of ***Raf***^***926***^ mutants on Dorsal nuclear localization

**Figure 5:**
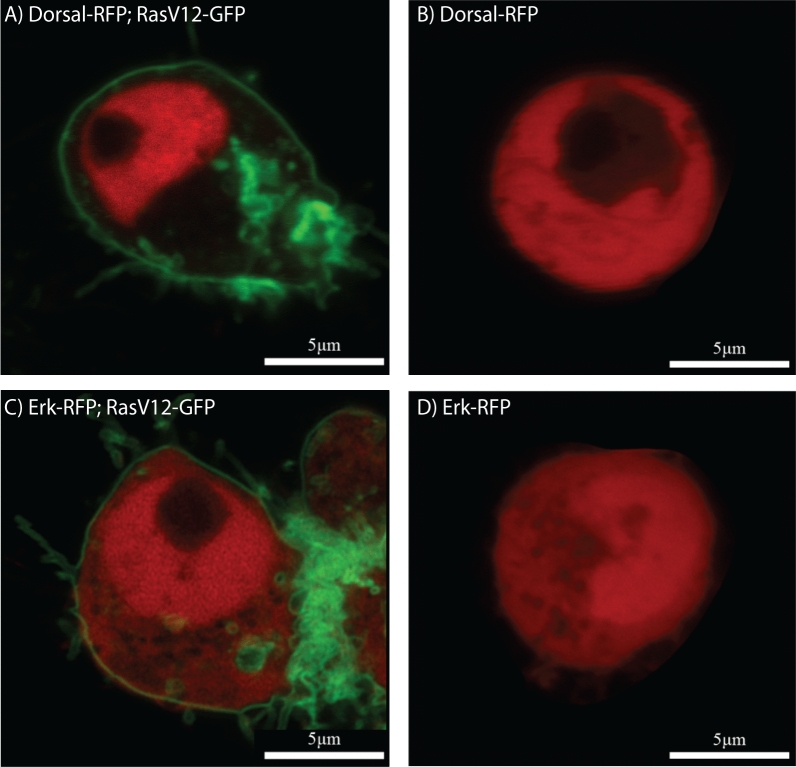
Dorsal and Erk localization in vitro. (**A**) Nuclear localization of Dorsal (red) in S2R+ cell expressing the gain of function Ras^V12^ (green). (**B**) Nuclear exclusion of Dorsal in S2R+ cell in the absence of RasV12 expression. Ubiquitous expression of Erk (red) in both cytoplasm and nucleus of S2R+ cell both (**C**) in the presence and (**D**) absence of Ras^V12^ (green). The black spot in the nucleus in (**A**–**C**) is the nucleolus.

**Figure 6:**
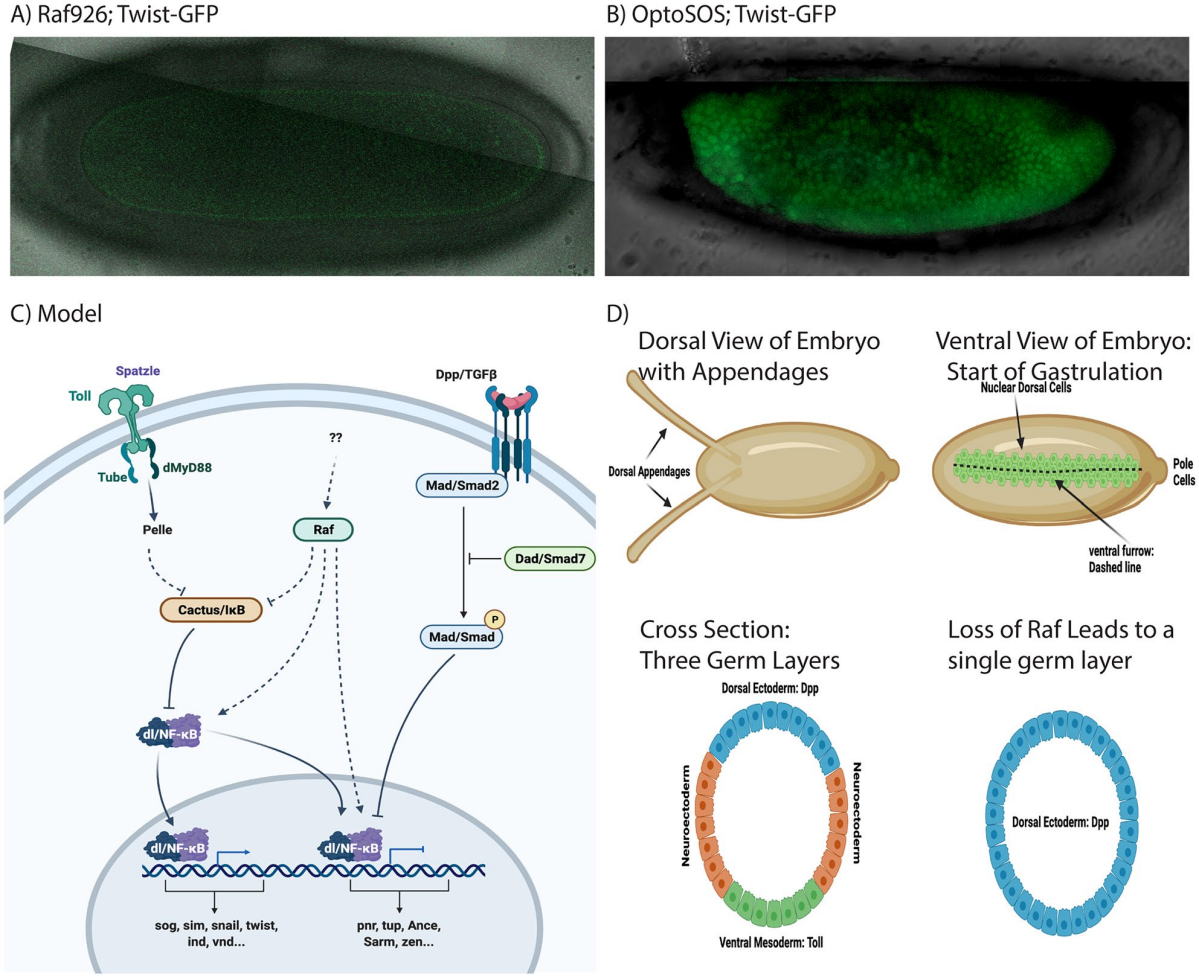
Opto-SOS drives Twist nuclear localization. (**A**) Live, confocal imaging of Twist-GFP expression in a *Raf*^*926*^ embryo shows no expression. (**B**) Activation of Raf using Opto-SOS showed uniform Twist-GFP (**C**). A model for Raf in Dorsal/Ventral signaling. (**D**) Schematic representation of the Dorsal and Ventral sides of Drosophila embryos with dorsal appendages and ventral furrow cells highlighted followed by cross section views of the early germ layers as defined in wildtype and Raf mutants. Figures were drawn on Biorender.com.

Based on the lack of a ventral furrow and the loss of Dorsal induced genes, we turned to D/V patterning pathways to determine the mechanism through which Raf is required for ventral furrow formation. The lack of ventral furrow in *Raf*^*926*^ was very similar to *dorsal* and *Toll* mutants, so we evaluated the activity of the Toll pathway in mutant embryos. The Dorsal protein localizes to the nucleus in a dynamic manner in response to Toll signaling in the ventral cells of pre-gastrulation embryos (Fig. 4A–C,G–I). *Raf*^*926*^ embryos showed a complete exclusion of Dorsal protein from the nucleus in both projection view (Fig. 4D–F) and in cross-section view (Fig. 4J–L), compared with the expected gradient of nuclear localization seen in wild-type embryos in both projection view (Fig. 4A–C) and in cross-section view (Fig. 4G–I). To observe this process in living embryos, we used Dorsal-GFP to image the early stages of Toll pathway activation. In normal development, a gradient of nuclear Dorsal-GFP can be observed before the ventral furrow forms, and the cells at the center of this gradient invaginate to begin ventral furrow formation (Combined Video 3, Videos 8 and 9, Still Images in S. Fig. 5A–E, S. Fig. 6A–E)^85^. We introduced the Dorsal-GFP construct into *Raf*^*926*^ embryos, which showed no discernible nuclear localization on the ventral side (Combined Video 3: Videos 10, 11, 12, Still Images in S. Fig. 5A′–E′,A″–E″ and S. Fig. 6A′–E′). The lack of nuclear Dorsal protein suggested that the dorsalized phenotype was due to a lack of Toll pathway activity despite many pathway components being upregulated (Fig. 2C).

As Raf is not believed to be a Toll pathway component, this finding was unexpected, and suggested genetically that a functional Raf allele was required for ventral Toll pathway activation. This led to a subsequent question: was it also sufficient? We expressed a gain of function Raf transgene in *Raf*^*926*^ embryos to investigate whether Raf alone could induce Dorsal nuclear localization. We observed that expression of Raf^GOF^ (Raf with a constitutively active kinase domain^40^) led to ubiquitous nuclear Dorsal protein in all cells of later embryos (Fig. 4P–R), shown in close-up (Fig. 4P′–R′). No nuclear Dorsal was observed in gastrulating *Raf*^*926*^ embryos not expressing Raf^GOF^ (Fig. 4M–O and in close-up M′–O′). The effect was difficult to observe in pre-gastrulation embryos due to the delay in expression of the GAL4/UAS system, but in later stages the data suggested Raf activity was sufficient to induce Dorsal nuclear localization. This finding was recapitulated in vitro, where in a cell culture model, we verified our in vivo findings in a tightly controlled environment. We designed two signaling sensors, the traditional MAPK cascade target ERK fused to RFP as the control for EGF signaling, and Dorsal fused to RFP as the indicator for Toll signaling. Expression of an activated Ras^V12^ was sufficient to induce both Dorsal and ERK nuclear localization (Fig. 5A–D).

### Raf is required for twist expression and mesoderm development

Ventral furrow cells form the mesoderm after invagination. This process requires an epithelial to mesenchymal transition which is mediated by the *twist* and *snail* genes^86,87^. Twist is first expressed within the presumptive mesoderm located on the ventral side of the embryo^87–90^. It acts with Dorsal to establish the presumptive mesoderm, and once mesoderm differentiation begins, Twist expression is maintained. Twist and Snail then drive mesoderm formation^90,91^. In our transcriptional analysis we observed that the *Raf*^*926*^ mutation showed a significant decrease in the expression of both (Fig. 3A).

To fully characterize the effect of Raf on ventral furrow formation, we next looked at the expression of Twist using a live imaging approach. Since the mesoderm is established by ventral cells, Twist in this case acts as a ventral mesoderm marker that gives us insight on how abnormal ventral development in early stages can disrupt late developments of the mesoderm. In a wildtype embryo (Combined Video 4: Video 13, Still Images in S. Fig. 7A–D) the ventral cells developed properly during cellularization and gastrulation, leading to ventral furrow formation and normal mesoderm development, as displayed by twist-GFP’s patterning of the late mesoderm. By contrast, *Raf*^*926*^ embryos lacked twist-GFP signal showing only yolk autofluorescence (Combined Video 4: Videos 14, 15, Still Images in S. Fig. 7A′–D′,A″–D″).

### Early embryonic EGF activation through optogenetics

As seen in the Raf^GOF^ experiments, activating EGF signaling in very early stages of embryogenesis was not possible using the standard Gal4/UAS system. We took advantage of a recently developed model of EGF signaling which uses an optogenetically activatable allele of SOS^92^. A major advantage of Opto-SOS is that expression of the allele alone does not affect flies if they are kept in the dark, so expression can be driven maternally in the F2 generation leading to very early EGF activation. In other words, adult flies expressing Opto-SOS can be obtained as long as they are not exposed to light meaning that eggs laid by Gal4/UAS-Opto-SOS flies will contain maternally deposited mRNA allowing very early expression and activation of SOS before gastrulation by application of blue light (488 mn laser). This has allowed Opto-SOS to be used to study patterning, transcription and morphogenesis^93,94^. A major finding of this work was that hyper-activation of the EGF pathway led to fate switching depending on cumulative ERK activity. Compressive forces moved yolk within the gastrulating embryo leading to popping, or ejection of yolk from the interior of the embryo to the exterior^93^. We used a combination of Gal4 drivers combining a maternal tubulin driver with a constitutive zygotic driver (matαTub-Gal4VP16 and Daughterless-Gal4) to drive early expression of Opto-SOS. We found that *Raf*^*926*^ embryos also showed yolk ejection to the exterior making these embryos very difficult to image at later stages (Combined Video 5, Videos 16–21), and required special preparation for cuticle preparations to remove the unused yolk (Fig. 1). Taking the gain-of-function and loss-of-function findings together suggests that ERK activity is required for a variety of force generating cellular processes during early gastrulation.

The loss of Raf led to a loss of ventral cell fates and to a loss of Twist expression as observed in both RNA-seq (Fig. 3B) and live imaging (Fig. 6A). Activation of Raf through expression of OptoSOS can also inhibit ventral furrow formation through activation of *huckebein* and *WntD*^95^, which we observed to be strongly downregulated in *Raf*^*926*^ embryos (Fold Expression Change log_2_ − 6.00 and − 8.95 respectively, Supplementary Table 1). In contrast to the loss of function condition (S. Fig. 7), OptoSOS expression activated Twist-GFP expression in all cells of the ectoderm (Fig. 6B). These findings taken together with^95^ suggest that Raf hyper activation and loss both affect ventral furrow formation.

## Discussion

Previous studies have revealed the multiple roles of Raf in developing the body axes of *Drosophila*. During oogenesis, Raf functions through Pipe in establishing dorsoventral fates of the egg. Targeted Raf activation in follicle cells is sufficient to dorsalize the eggshell whereas reduced Raf ventralizes the eggshell^40^. During embryogenesis, Raf defines terminal structures and establishes ventral fates of the neuroectoderm. Early maternal/zygotic mutant screens identified *Raf* (*pole hole*) mutants as lacking terminal structures and lacking visible ventral cuticle especially in amorphic alleles inducing mutations in conserved region 1 (Fig. 1A)^17,18,64^. Although previous studies have comprehensively screened and characterized many Raf mutants, no connection between Raf and ventral fate determination has been shown during embryonic development (Fig. 6C,D). We applied a modern screening approach, where a series of mutations were made and characterized through whole genome sequencing^63^, and found a strong mutation in Raf that led to a complete loss of ventral structures. We characterized the *Raf*^*926*^ embryo and established its dorsalized phenotype. We discovered that this phenotype was not due to defective oogenesis and could not be explained completely by defective *torso* signaling.

Most importantly, this Raf function appears to be independent of the MAPK cascade, as there is no evidence of active MAPK in ventral cells. Studies looking at phosphorylated MAPK show activation at the anterior and posterior poles in early stages, lateral activity in early gastrulation and activation in the mesoderm^81^. Interaction of MAPK and Toll pathway has been observed at early stages through Capicua and WntD^96,97^. This suggests that Raf’s function in the most ventral cells is not through MAPK, but could perhaps function through direct phosphorylation of IκB/Cactus^98^ (Fig. 6C) as previous studies into the oncogenic properties of Raf showed that, in mammalian cell culture, Raf regulates NFκB signaling directly by binding and phosphorylating IκB^99^. Raf could act similarly to MAP3K14, also known as NIK (NF-κB inducing Kinase), which cooperates with IKKα in mammals to phosphorylate IκB, thereby activating NF-κB signal^49,50,100^. As Raf is a MAP3K, it could play a similar role in *Drosophila* Toll signalling, but additional investigations into the precise molecular mechanism connecting Raf and the Toll pathway will be required. Alternatively, Raf could function in conjunction with Sterile 20 like kinase in a MAPK-independent, parallel pathway^60,61^.

In short germband insect species, EGF signaling helps to establish dorsoventral polarity^101^, and our results suggest a role for the EGF signaling component Raf in the long germband insect *Drosophila*. EGF signaling is involved in patterning of the oocyte where signaling from the egg to the follicle cells establishes egg polarity^30,102^. In the embryo, EGF’s dorsal–ventral patterning activity is thought to be limited to the neuroectoderm. Both the Toll and EGF pathways are highly conserved in oncogenesis and immune function, and this study presents the first in vivo evidence that EGF and Toll signaling are connected in a normal physiological/developmental system^103–106^. Further characterization of signalling events bridging EGF and Dorsal/NF-κB signalling could yield valuable insight into the regulation of the therapeutically important NF-κB family of proteins and broaden our understanding of how neoplasms which have attained EGF-family mutations interface with the body’s immune surveillance mechanisms.

Limitations of the study: there is a major caveat to this study, in that this this work relies on the *Raf*^*926*^ allele of *Raf,* a novel mutant with phenotypes not previously reported. Although we show that overexpression of the truncated *Raf*^*926*^ fragment tagged with mCherry did not show any obvious dominant negative effects, it is not possible to rule out completely that there are dominant effects of the truncated *Raf*^*926*^ due to possible interference of the mCherry tag. Further, *Raf*^*926*^-mCherry was expressed in an otherwise wild-type *Raf* background, and although duplication mapping did not show lethal second site mutations, it remains possible that non-lethal mutations exist on the chromosome that affect dorsal–ventral patterning. We did attempt to expand the study to other novel *Raf* mutants, but these did not produce embryos, suggesting further studies will be required to understand Raf’s role in embryogenesis by defining true nulls and the mechanism by which the localization of Dorsal is affected by Raf.

## Methods

### Crosses and expression of UAS construct

Maternally mutant eggs were generated by the dominant female sterile technique where balanced mutants are crossed to the dominant female sterile mutation *Ovo*^*D2*^ and recombination is induced using the FLP/FRT method in ovaries^66,107^. The *Ovo*^*D2*^, FRT19A double mutant line was generated by recombining X chromosomes with *Ovo*^*D2*^ and FRT19A in a female rescued for sterility by a second chromosome duplication carrying a wildtype *Ovo* gene (Dp(1;2)w + 64b). Oregon R was used as the wild-type strain. Please see Flybase for further details on mutants used (flybase.bio.indiana.edu). Mutants used: *Raf*^*926*^. For mis-expression experiments, we used a GAL4 driver combining the early expression of the matαTub-Gal4VP16 together with daughterless-Gal4 on the third chromosome (matda-gal4). All X-chromosome mutants use FRT19A. The following crosses were conducted.

1. *w*, *Raf*^*926*^, FRT19A/FM6; matda-gal4/+female x *w*, *ovo*^*D2*^, FRT19A/male
2. *w*, *Raf*^*926*^, FRT19A/*Ovo*^*D2*^, FRT19A female x FM6/male
3. *w*, *Raf*^*926*^, FRT19A/*Ovo*^*D2*^, FRT19A; matda-gal4/+female x UAS-Raf^926^-mCherry, attP2
4. *w*, *Raf*^*926*^, FRT19A/ *Ovo*^*D2*^, FRT19A; matda-gal4/+female x UAS-Raf^GOF^^40^
5. *w*, *Raf*^*926*^, FRT19A/*Ovo*^*D2*^, FRT19A; Dorsal-GFP/+x w; Dorsal-GFP^85^
6. *w*, *Raf*^*926*^, FRT19A/*Ovo*^*D2*^, FRT19A; Twist-GFP/+x w; Twist-GFP^108^
7. *w*, *Raf*^*926*^, FRT19A/*Ovo*^*D2*^, FRT19A x QUAS-GFP, Tubulin-QF2^109^
8. *w*, *Raf*^*926*^, FRT19A/*Ovo*^*D2*^, FRT19A; matda-gal4/+female x Zcl-GFP^110^
9. *w*, *Raf*^*A*^, FRT19A/ *Ovo*^*D2*^, FRT19A^63^
10. *w*, *Raf*^*B*^, FRT19A/ *Ovo*^*D2*^, FRT19A^63^
11. nanos-gal4; UAS-Cas9 female x Raf TRiP- KO guide RNA^70^
12. matda-gal4/UAS-OptoSOS female x w; UAS-OptoSOS^92^
13. matda-gal4/UAS-OptoSOS female x UAS-OptoSOS; Twist-GFP^108^
14. matda-gal4/+female x UAS-Raf^926^-mCherry, attP2 (This study)

For early embryogenesis imaging, the GFP tagged gene was provided both maternally and zygotically to begin expression at the earliest possible stage of embryogenesis. For example, Dorsal-GFP could be observed in the nuclei of cells about to form the ventral furrow (Video 8), but Twist-GFP which is expressed when cells transition to mesoderm could only be observed after ventral furrow formation (Video 13).

X chromosomes were marked with *w*^*1118*^ mutation and the CyO and FM6 balancers were marked GFP to simplify analysis. As mothers were heterozygous for the Gal4 source, maximal rescue is reflected by a drop of phenotype to 50% (only half of the embryos will express Gal4). For all crosses, more than 100 embryos were analyzed in multiple, separate experiments (n > 95). Additional stocks were obtained from the Bloomington stock center including the Q system^111^.

### Mutant, transgene and driver lines

The mutation was genetically mapped on the chromosome X by complementation with deletion/duplication strains, specifically using molecularly defined P[acman] duplications to narrow the source of lethality on the X chromosome^112,113^. It was precisely localized to 3A1-A2 through suppression by P[acman] duplication DC107 which covers Raf’s protein coding exons or the RA splice variant, but not the non-coding regions in the RE splice variant^67^. Additional confirmation was obtained by crossing *Raf*^*926*^ (genetically rescued males carrying the DC107 duplication) to molecularly defined Raf mutations *Raf*^*A*^ and *Raf*^*B*^, which did not complement the lethality. The molecular nature of the *Raf*^*926*^ allele with a deletion of 17 nucleotides (GAGTACGACTATGTGAT) from the 479th nucleotide of the protein-coding region, was found by Sanger sequencing. The genomic DNA was amplified by PCR, sequenced, and the products were cloned into pENTR vectors (Invitrogen) and recombined using Gateway technology (Invitrogen) into pUASg.AttB.mCherry vectors for fly injection^114,115^. The DNA was injected into strains P[CaryP]attP2 68A4 and P[CaryP]attP40 25C6 by BestGene Inc California^116^. For constitutive expression in S2R+ cells, the pDONR vectors were recombined into the Gateway destination vector, pAW (Drosophila Gateway Vector collection, Carnegie Institution), with NH2 terminus flag-tag for Raf, Raf^926^ and Raf^GOF^, HA-tag for Ras, Ras^V12^ and Ras^N17^ and COOH-terminus RFP and GPF-tags for Erk, dorsal and Ras^V12^ constructs used in live imaging. The Toll and Dorsal optogenetic constructs were obtained by gene synthesis and combined with cryptochrome 2 and mCherry cDNA as described in^117^.

Additional genetic testing was done with two further novel Raf point mutants, *Raf*^*A*^ (H546Y) and *Raf*^*B*^ (L163R) obtained from the Bloomington Drosophila Stock Center. CRISPR/Cas9 studies were carried out using Raf TKO.GS00615 gRNA line in combination with a *nanos* Gal4 driven UAS-Cas9^70^.

### Immunofluorescence

Embryos were fixed with Heat-Methanol treatment^118^ or with heptane/4% formaldehyde in phosphate buffer (0.1 M NaPO4 pH 7.4)^119^. The antibodies used were: 1:10 – 1:100 anti-dorsal (mouse mAb, 7A4, Developmental Studies Hybridoma Bank (DSHB) developed under the auspices of the NICHD and maintained by The University of Iowa, Department of Biological Sciences, Iowa City, IA 52242), 1:500 Hoechst 33342 for nuclear staining. Staining, detection and image processing as described in^120^.

### Light-sheet microscopy

Embryos at cellularization stage and earlier (Stages 1–5) were selected using halocarbon oil (Sigma). Embryos were carefully dechorionated using bleach, rinsed twice with water and dried, prior to loading into a capillary filled with 1% low-melting agarose (Sigma). Embryo(s) were then carefully oriented under a dissecting microscope using a thin piece of wire and metal probe such that the embryo was upright with the anterior–posterior axis aligned with the axis or perpendicular to the axis of the glass capillary. The agarose was pushed out of the capillary and the sample was suspended freely in the water-filled sample chamber of the Lightsheet Z1 microscope and imaged with a water immersion objective at 20×.

### Scanning electron microscopy

Formaldehyde fixed embryos (with slight adjustment to the cited method: overnight at 4 °C with rocking; 8 times post-fixation methanol washes) were washed once and re-hydrated with phosphate buffer for 10 min with rocking. Embryos were then applied to a microscopy slide. Phosphate buffer on microscopy slide was removed as much as possible. The slide with embryos was then dried prior to imaging. Imaging was performed with Hitachi TM3030Plus table-top scanning electron microscope at 1000×.

### RNA preparation and RNA-sequencing

Mixed stage embryos, 0–12 h after deposition at 18 °C, were dechorionated in bleach, washed in water and 100% ethanol prior to RNA extraction using the ISOLATE II RNA Mini Kit’s protocol (Bioline, UK). The extracted RNA was quantified using Nanodrop (Thermo Fisher Scientific). Library preparation was performed using 1 µg of total RNA and sequencing was performed using Illumina HiSeq 4000 System (2 × 151 bp read length, 40 million reads per sample) by NovogeneAIT Genomics (Singapore). Three biological replicates were sequenced for each condition.

#### RNA-seq analysis

##### Data processing and QC

RNA-seq was aligned against BDGP6.22 (Ensembl version 97) using STAR v2.7.1a^121^ and quantified using RSEM v1.3.1^122^. Reads annotated as rRNA, snoRNA, or snRNA were removed. Genes which have less than 10 reads mapping on average over all samples were also removed. Differential expression analysis was performed using DESeq2^123^. Pairwise comparisons were performed using a Wald test, with independent filtering. To control for false positives due to multiple comparisons in the genome-wide differential expression analysis, the false discovery rate (FDR) was computed using the Benjamini–Hochberg procedure. For the re-analysis of the RNA-seq dataset from Koenecke et al*.*, all of the above is the same except the version of STAR used was v2.9.1a^121^. The gene-level counts were transformed using a variance-stabilizing transformation, converted to z-scores, and clustered using k-means. The total within-cluster sum of squares distances and the elbow criterion was used to determine the optimal number of clusters (k = 3) in this dataset. The significance of the overlap between the RNA-seq data from Koenecke et al*.* and our dataset was computed using random sampling without replacement. After randomly sampling the respective groups of genes, the numbers of overlaps between the two datasets were plotted on a histogram to visualize the probability distribution. The observed number of overlaps was then compared to this distribution and a p-value was calculated.

##### Functional enrichment analysis

For the analysis of genes upregulated and downregulated in *Raf*^*926*^ mutants (Fig. 2c), Gene Ontology (GO) and KEGG pathway enrichments were performed using EnrichR^124–126^. The GO term and KEGG pathway enrichments of the germ layer clusters from the Koenecke et al. RNA-seq dataset were also performed with EnrichR. Terms with an FDR < 10% were defined as significantly enriched. The gene sets used in the gene set enrichment analysis (GSEA) were obtained from MSigDB^127–129^ via the msigdbr package, and the analysis itself was carried out using clusterProfiler^130^.

### Transfection and protein quantification

Drosophila S2R+ cells were maintained in Schneider’s Drosophila Medium with 10% Fetal Bovine Serum and 1% Penicillin and Streptomycin (Invitrogen). Cells were plated at 80% confluence on 60 mm dish 24 h prior to transfection with relevant constructs using the Effectene Transfection Reagent (Qiagen) according to manufacturer’s instructions. Untransfected cells were used as negative control.

### Live cell confocal microscopy

S2R+ cells were plated on 35 mm glass-bottom dish at 80% confluence and transfected as mentioned. Cells were imaged 24 h after transfection using the Zeiss LSM800 confocal microscope with 63× oil immersion lens. Cells transfected with either Dorsal or Erk were used as negative controls for respective experiments. For live embryo imaging, embryos were processed as for Lightsheet microscopy and mounted in 1% low melting point agarose on glass bottomed petri dishes. Imaging was done on a Zeiss LSM 800 (Carl Zeiss, Germany) using the following settings: 1% laser power for 488 nm; 5% laser power for 561 nm. Images were processed using the ZEN 2014 SP1 software (Carl Zeiss, Germany).

## Supplementary Information

Supplementary Legends.

Supplementary Video 1.

Supplementary Video 2.

Supplementary Video 3.

Supplementary Video 4.

Supplementary Video 5.

Supplementary Figure 1.

Supplementary Figure 2.

Supplementary Figure 3.

Supplementary Figure 3.

Supplementary Figure 4.

Supplementary Figure 5.

Supplementary Figure 6.

Supplementary Figure 7.

Supplementary Table 1.

## Data Availability

RNA-seq data from this study has been deposited to GEO (GSE178187). Further information and requests for resources and reagents should be directed to and will be fulfilled by the Lead Contact Nicholas S. Tolwinski (Nicholas.Tolwinski@Yale-NUS.edu.sg).

## Materials availability

All unique reagents generated in this study are available without any restriction.

## Code availability

All code necessary to recreate the results from the RNA-seq analysis presented is available from: https://github.com/harmstonlab/Raf926.
