# Supplementary material for: A non-canonical Raf function is required for dorsal–ventral patterning during Drosophila embryogenesis
Source: Sci Rep. 2022 May 10;12:7684. doi: 10.1038/s41598-022-11699-3 (PMC9090920; doi:10.1038/s41598-022-11699-3)
Supplement: Supplementary file 1 — Supplementary Legends. [file 41598_2022_11699_MOESM1_ESM.docx]

**Video Legends:**

**Combined Video 1. Live imaging of wildtype and *Raf^926^* embryos.**

**(Video 1)** Lightsheet imaging showing that Raf^926^ overexpression (double driver: Arm-Gal4, Daughterless-Gal4>UAS- Raf^926^-mCherry) in an otherwise wildtype embryo does not affect embryonic development.  **(Video 2)** Lightsheet imaging showing severe defects in *Raf^926^* embryo expressing Tubulin-QF2>QUAS-GFP and Daughterless-Gal4>Myr-Tomato. **(Video 3)** Development is severely affected as shown by Utrophin-GFP and Histone-RFP lightsheet imaging of *Raf^926^* embryo.  **(Video 4)** Normal embryonic development as shown by Utrophin-GFP and Histone-RFP lightsheet imaging of an otherwise wildtype embryo.

**Combined Video 2. Brightfield imaging of embryos from cellularization to early gastrulation.**

**(Video 5)** Normal cellularization to ventral furrow development in a wildtype embryo. **(Video 6)** Lack of ventral furrow formation in a *Raf^926^* embryo. (**Video 7)** Ventral furrow formation in a *bicoid, nanos, torsolike* triple mutant embryo, indicating that the absence of anterior to posterior patterning does not affect ventral furrow formation and is different form the *Raf^926^* phenotype.

**Combined Video 3. Confocal imaging of early embryos expressing Dorsal-GFP.**

**(Video 8)** Nuclear localization of Dorsal-GFP on the ventral side of a wildtype embryo expressing Dorsal-GFP. The cells along the ventral midline invaginate to form the ventral furrow. (**Video 9)** Provides a close-up view. (**Video 10)**  No discernible nuclear Dorsal-GFP is present in a *Raf^926^* embryo expressing Dorsal-GFP imaged from cellularization suggesting that the Toll pathway was not properly activated. (**Video 11)** Provides a close-up view. (**Video 12)** Brightfield imaging of the embryo in Video 10 to confirm that embryo was developing.

**Combined Video 4. Confocal imaging of late embryos expressing twist-GFP. (Video 13)** The twist-GFP pattern in the wildtype embryo expressing twist-GFP indicates normal mesoderm development as a result of proper early ventral furrow formation. (**Video 14)** The lack of twist-GFP signal in a *Raf^926^* embryo expressing twist-GFP indicates no proper mesoderm development as a result of the lack of early ventral cell fates and lack of ventral furrow formation. (**Video 15)** Brightfield imaging of the embryo in Movie 14 to confirm that embryo was developing.

**Combined Video 5. Confocal and brightfield imaging of later stage *Raf^926^* embryos.**

**(Video 16)** Brightfield imaging showing yolk movements in *Raf^926^*, **(Video 17)** GFP (Tubulin-QF2>QUAS-GFP) imaging of the same embryo and **(Video 16)** the composite. A second embryo with somewhat different yolk movements **(Video 19-21).**

**Supplementary Figures Legends:**

**Supplementary Figure 1. Cuticular preparations of embryos derived from RafTKO.GS00615 gRNA line combined with *nanos-Gal4* driven UAS-Cas9 showing dorsalized embryos.** (A) and (B) Embryos imaged using a 10X objective, darkfield. (C) and (D) Embryos imaged using a 20X objective, phase contrast. (E) and (F) Embryos imaged using a 40X objective, phase contrast. All embryos display dorsal hairs and do not produce ventral denticles.

**Supplementary Figure 2. Still images from Videos 5-7 (Combined Video 2). (A)** shows wildtype, *bnt (bicoid, nanos, torsolike)*, and *Raf^926^* germ-line clone embryos during cellularization. **(B-D)** show progression after cellularization. Wildtype and *bnt* embryos develop ventral furrows whereas *Raf^926^* embryos do not and instead undergo abnormal twisting and turning during gastrulation.

**Supplementary Figure 3: Analysis of RNA-seq from *Raf^926^* mutant embryos. (A)** Principal component analysis reveals a clear separation between WT and *Raf^926^* embryos, with the first principal component accounting for 92% of the variance. **(B)** Expression of *Raf* is significantly downregulated in *Raf^926^* embryos compared to WT showing less than half the read counts as expected for a maternal effect mutation with heterozygous zygotic expression **(C)** Multiple components of the Toll pathway (KEGG:dme04624) are upregulated in *Raf^926^* mutant compared to WT embryos.

**Supplementary Figure 4: The expression of key germ layer marker genes is affected in *Raf^926^* mutant embryos.** Expression changes of all **(A)** ectoderm, **(B)** neuroectoderm, and **(C)** mesoderm marker genes as proposed by [82] that have detectable expression in our model. **(D)** Re-analysis of RNA-seq data profiling from Knoecke *et al.* identifies three clusters which are associated with both distinct mutations in the Toll pathway and distinct germ layers. **(E)** GO Biological process enrichment analysis show that each cluster is enriched for processes associated with the corresponding germ layer (FDR<0.1). **(F)** Venn diagram highlighting multiple genes upregulated by Raf^926^ mutation overlap with those specific to the ectoderm cluster.

**Supplementary Figure 5. Still images from videos 8, 10, 12 show absence of nuclear Dorsal gradient in live developing *Raf^926^* embryos.** **(A-E)** Surface view of the ventral side of the wildtype embryo expressing Dorsal-GFP from cellularization **(A)** to ventral furrow formation **(B-E)**. Dorsal-GFP is localized in the nucleus. **(A’-E’)** Surface view of the *Raf^926^* embryo expressing Dorsal-GFP from cellularization **(A’)** to early gastrulation **(B’-E’)**, showing no discernible nuclear localization on the ventral side. **(A’’-E’’)** show the corresponding brightfield images of (A’-E’) indicating that the phenotype observed is from a live *Raf^926^* embryo.

**Supplementary Figure 6. Still images from videos 9 and 11 show no nuclear Dorsal in live developing *Raf^926^* embryos.** Close-ups of **(A-E)** surface view of the ventral side of the wildtype embryo expressing Dorsal-GFP from cellularization **(A)** to ventral furrow formation **(B-E)**. Dorsal-GFP is localized in the nucleus. **(A’-E’)** Surface view of the *Raf^926^* embryo expressing Dorsal-GFP from cellularization **(A’)** to early gastrulation **(B’-E’)**, showing no discernible nuclear localization on the ventral side.

**Supplementary Figure 7. Still images from Videos 13-15 show *Raf^926^* embryos fail to develop mesoderm. (A-D)** Late wildtype embryo expressing twist-GFP shows normal mesoderm development. **(A’-D’)** Late *Raf^926^* embryo expressing twist-GFP shows a lack of twist-GFP signal and no proper mesoderm development. **(A’’-D’’)** show the corresponding brightfield images of (A’-D’) indicating that the phenotype observed is from a live *Raf^926^* embryo.

**Supplementary Table 1: Comprehensive list of all gene expression changes discussed in the manuscript.**
