# Supplementary figures and images for: A non-canonical Raf function is required for dorsal–ventral patterning during Drosophila embryogenesis
Source: Sci Rep. 2022 May 10;12:7684. doi: 10.1038/s41598-022-11699-3 (PMC9090920; doi:10.1038/s41598-022-11699-3)

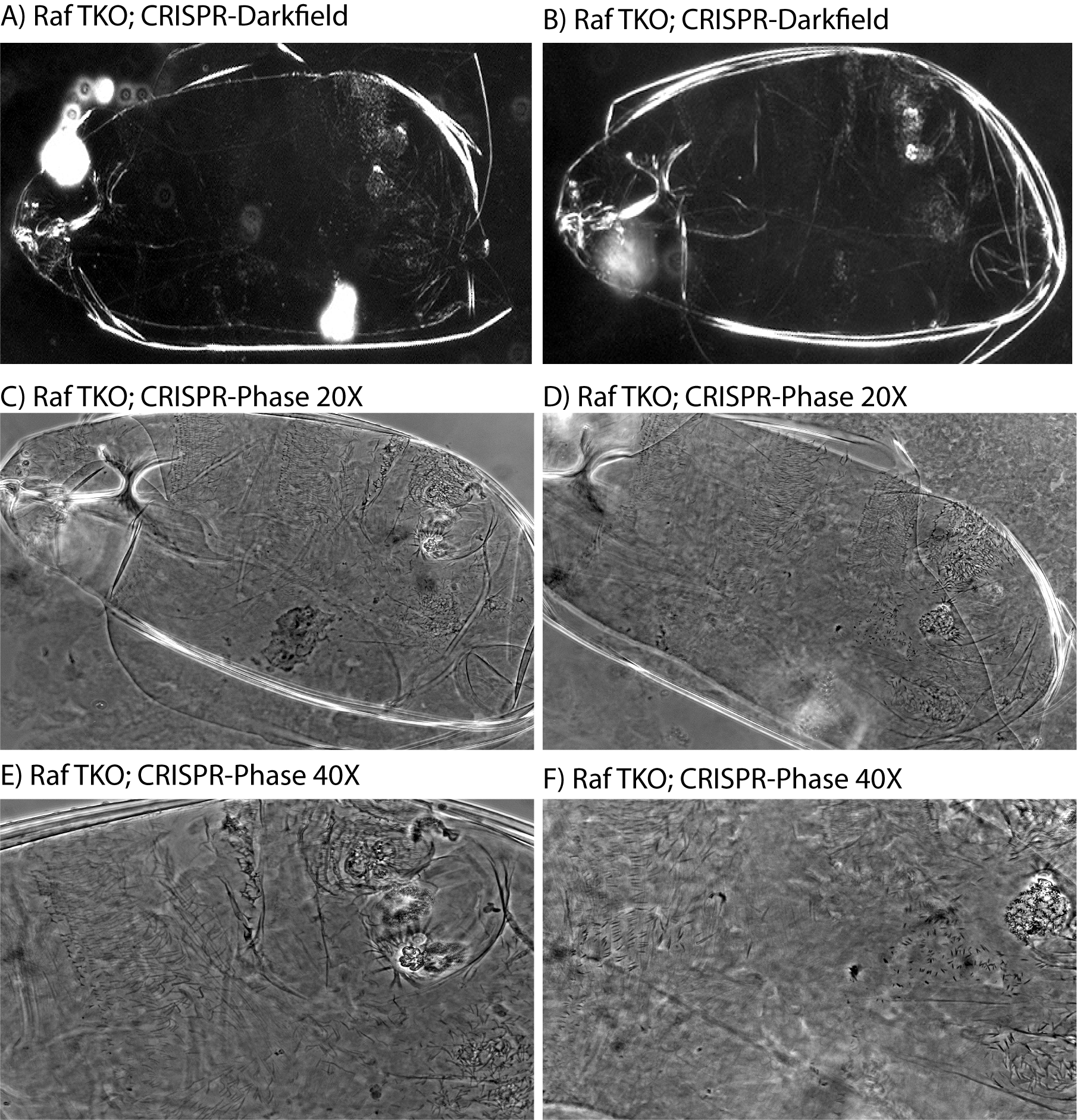

Supplement: Supplementary file 7 — Supplementary Figure 1. [file 41598_2022_11699_MOESM7_ESM.tif]

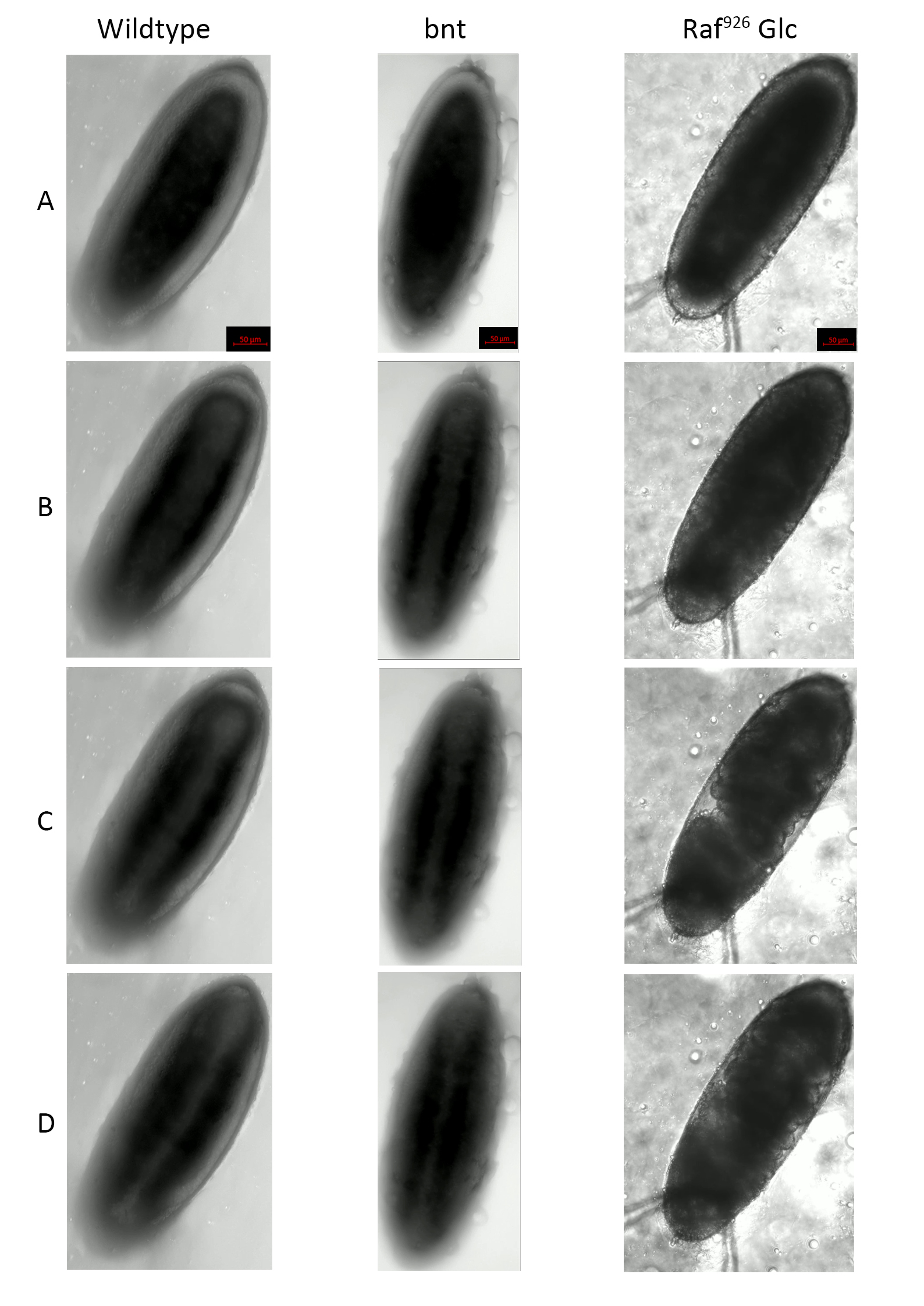

Supplement: Supplementary file 8 — Supplementary Figure 2. [file 41598_2022_11699_MOESM8_ESM.jpg]

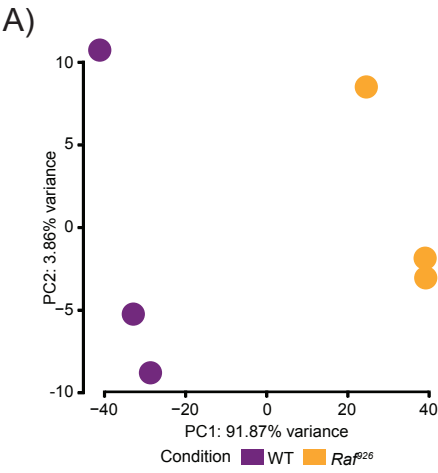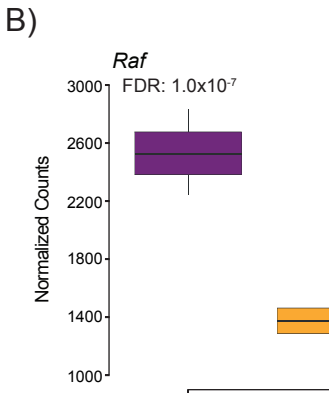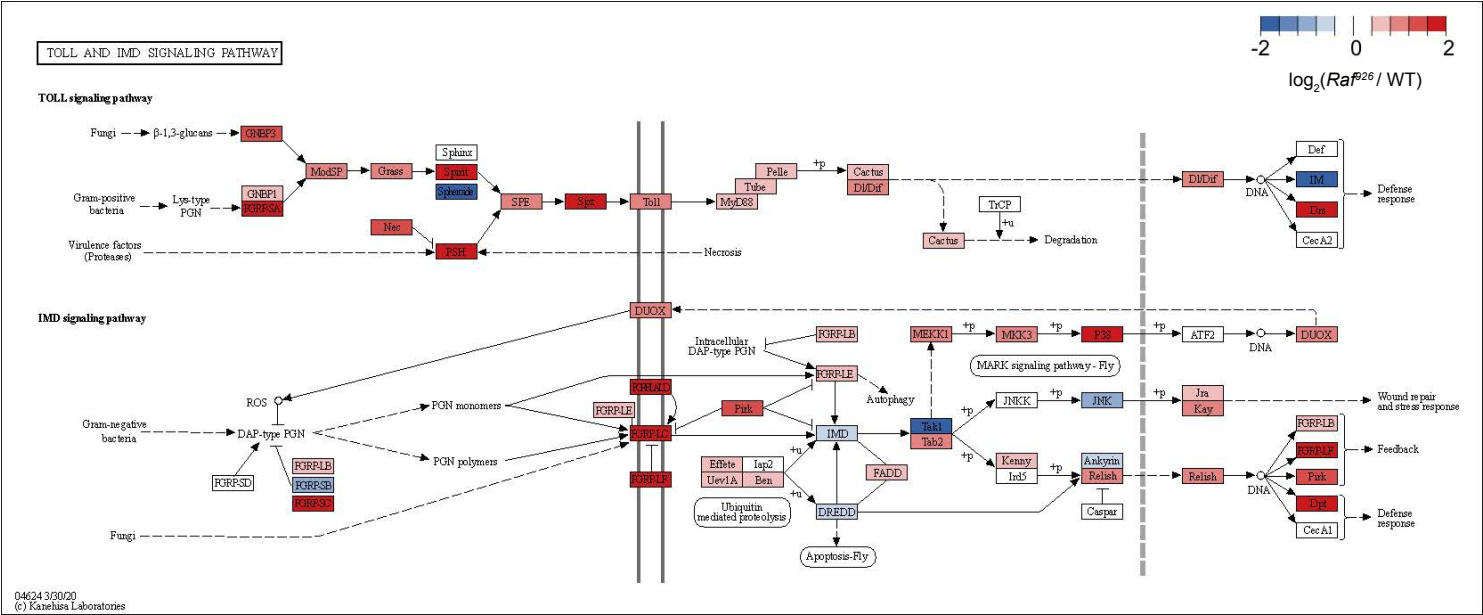

Supplement: Supplementary file 9 — Supplementary Figure 3. [file 41598_2022_11699_MOESM9_ESM.pdf]

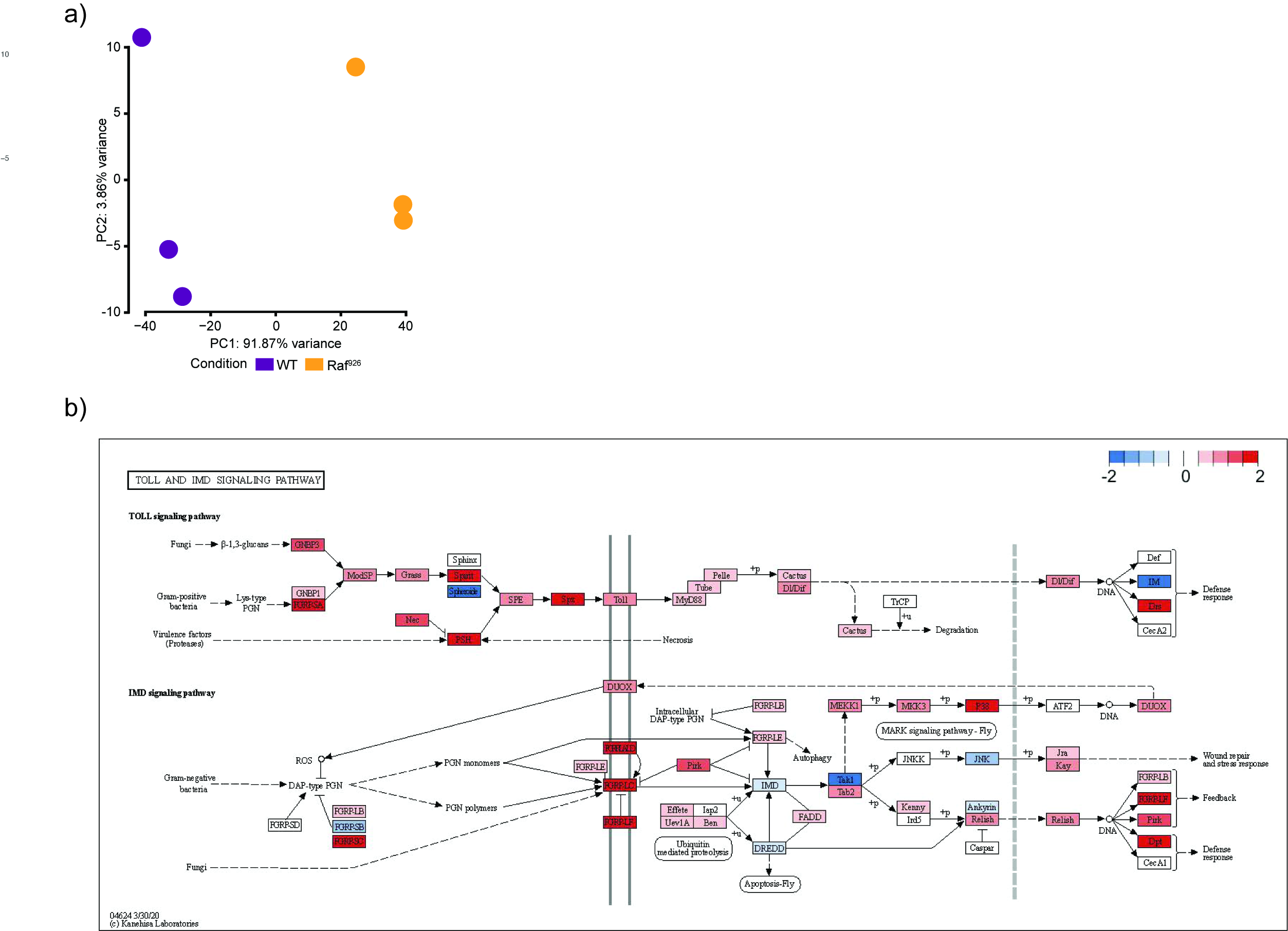

Supplement: Supplementary file 10 — Supplementary Figure 3. [file 41598_2022_11699_MOESM10_ESM.tif]

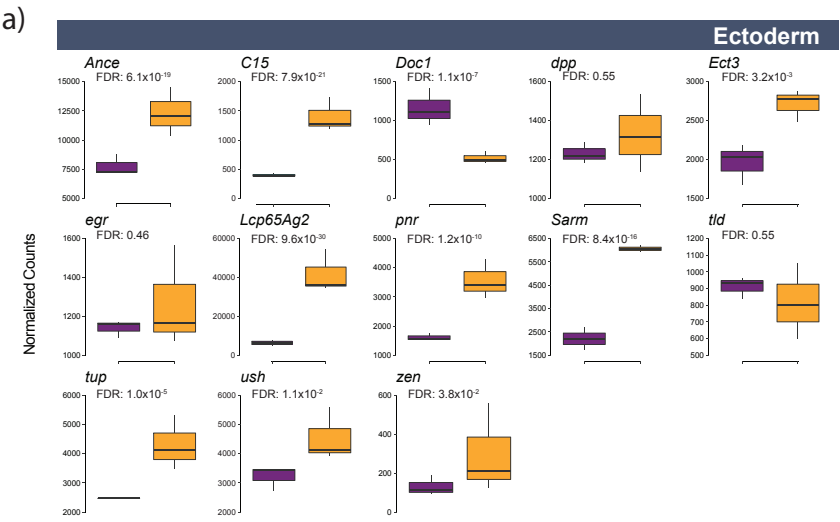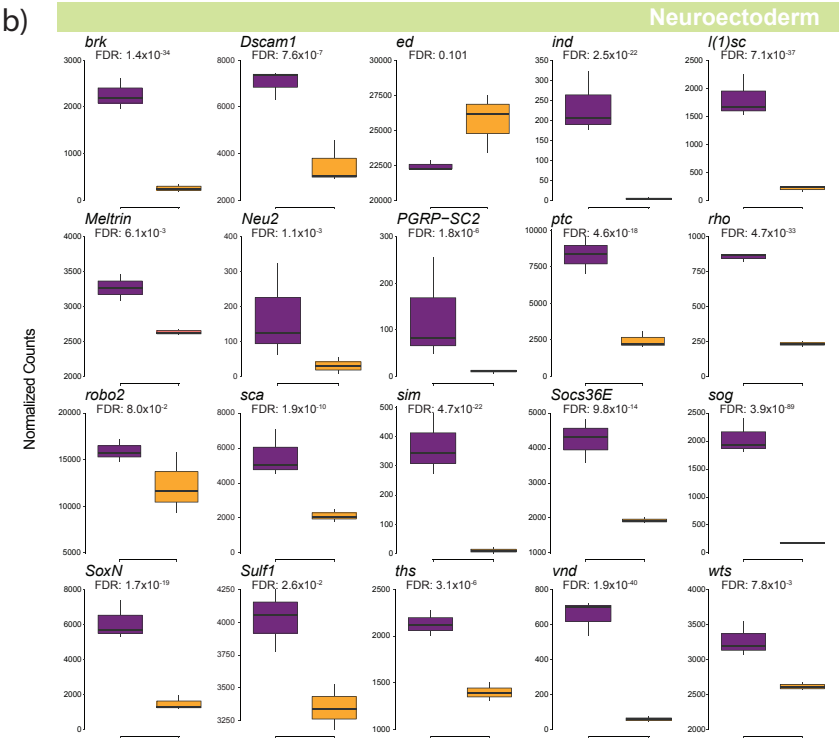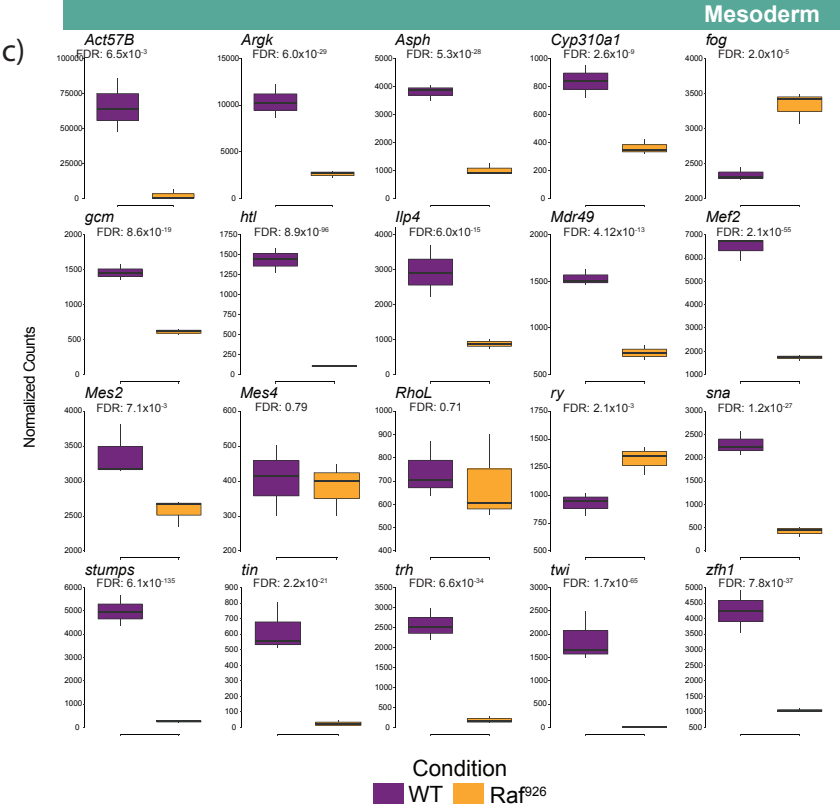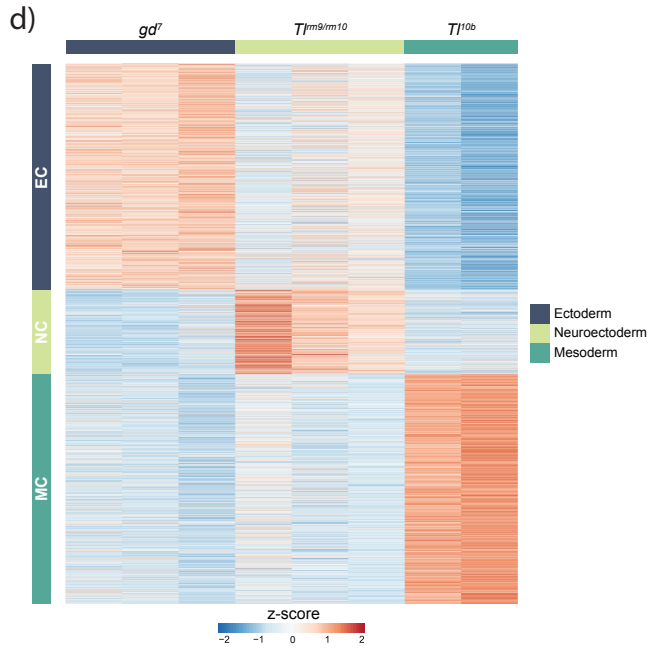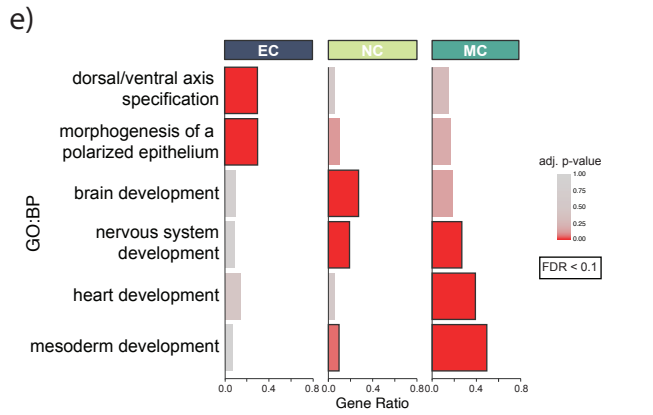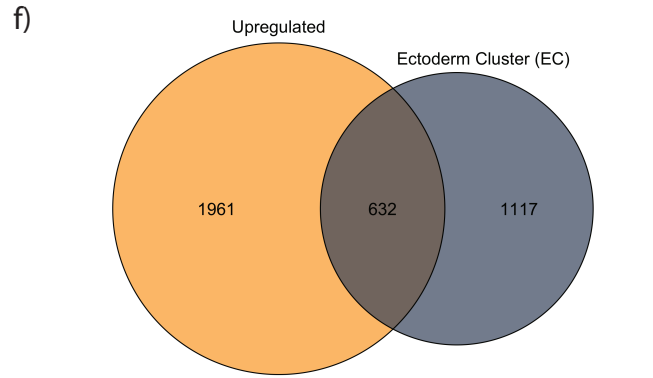

Supplement: Supplementary file 11 — Supplementary Figure 4. [file 41598_2022_11699_MOESM11_ESM.pdf]

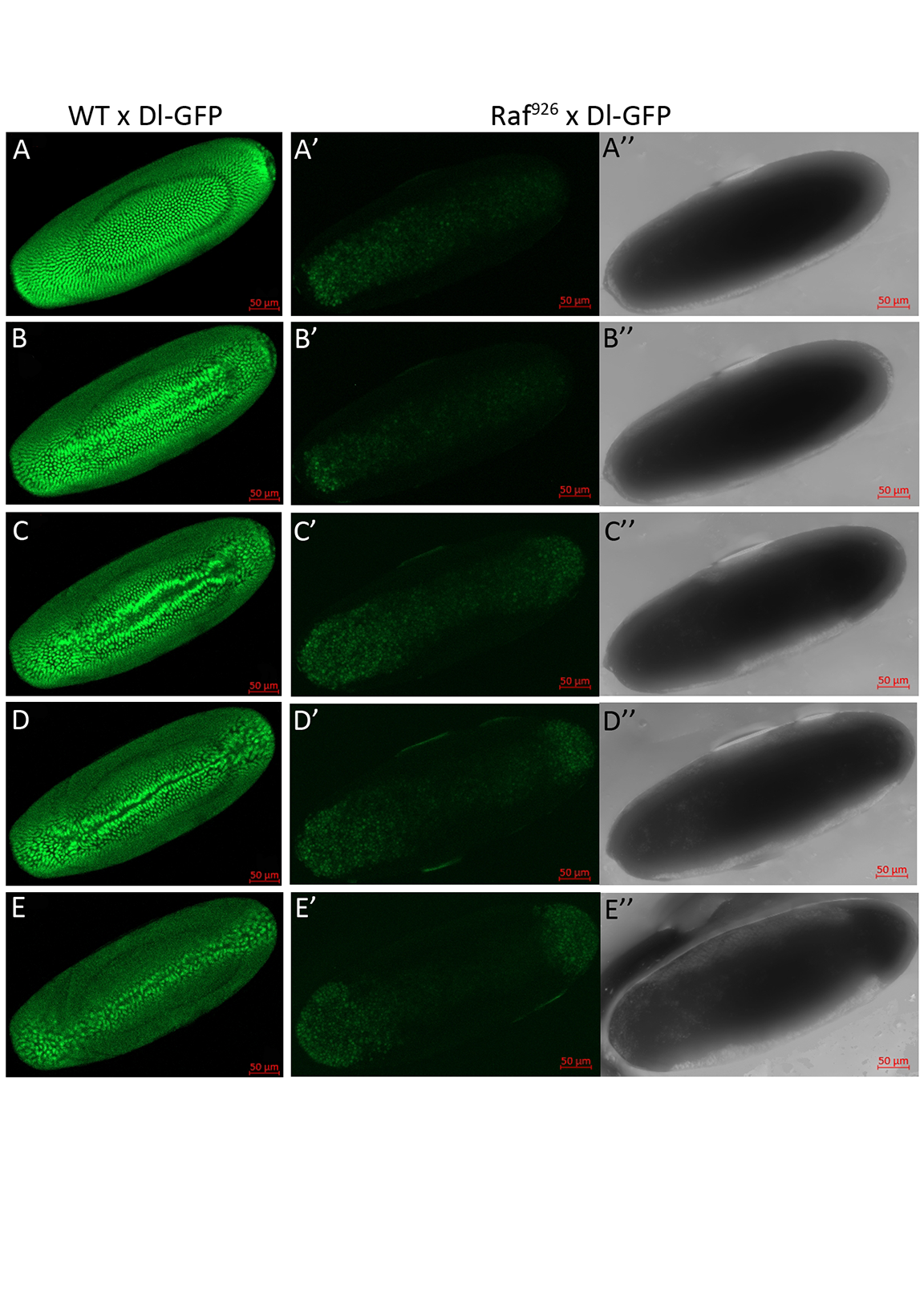

Supplement: Supplementary file 12 — Supplementary Figure 5. [file 41598_2022_11699_MOESM12_ESM.tif]

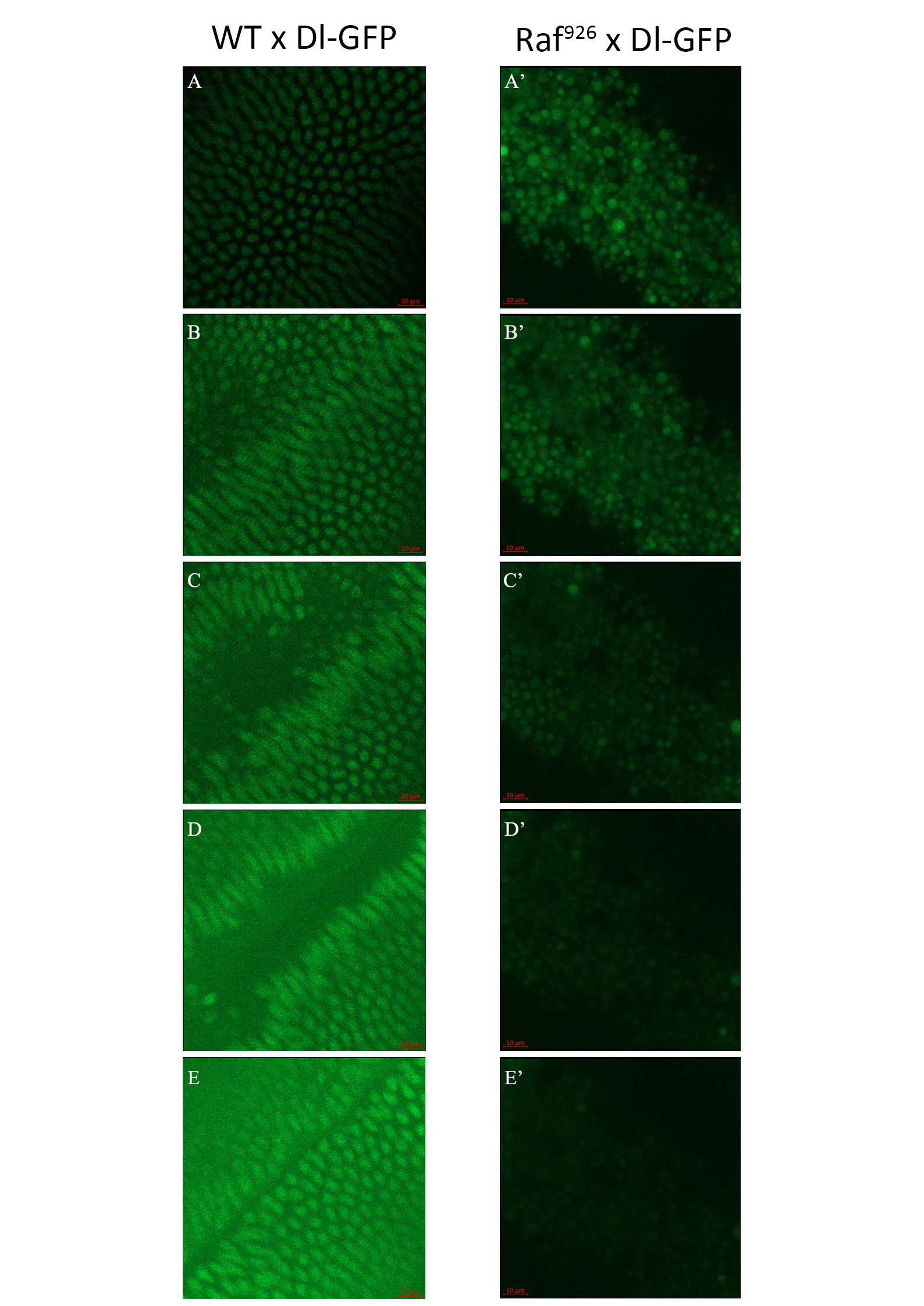

Supplement: Supplementary file 13 — Supplementary Figure 6. [file 41598_2022_11699_MOESM13_ESM.tif]

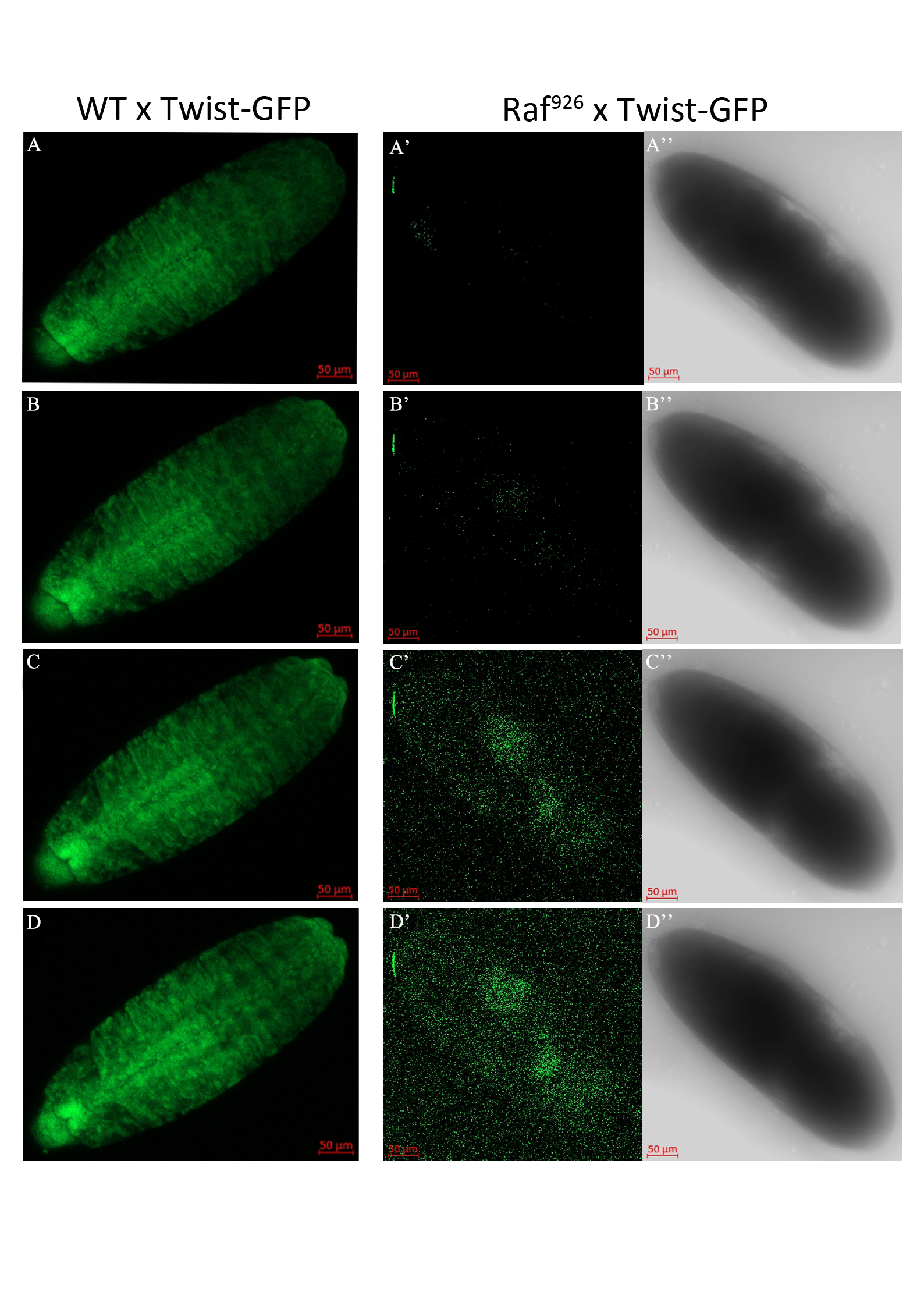

Supplement: Supplementary file 14 — Supplementary Figure 7. [file 41598_2022_11699_MOESM14_ESM.tif]
